# Head of Zebu cattle (*Bos Taurus indicus*): sectional anatomy and 3D computed tomography

**DOI:** 10.1186/s12917-024-04141-5

**Published:** 2024-07-16

**Authors:** Ahmed G. Nomir, Ashraf El Sharaby, Basma G. Hanafy, Mohamed M. A. Abumandour

**Affiliations:** 1 Department of Anatomy and Embryology, Faculty of Veterinary Medicine, Damanhour University, Damanhour, 22511 Egypt; 2 Department of Anatomy and Embryology, Faculty of Veterinary Medicine, Alexandria University, Post Box: 22758, Alexandria, 21944 Egypt

**Keywords:** Zebu cattle, Head, Anatomy and cross-sections, Paranasal sinuses, Conchal sinuses, 3D-CT reconstruction

## Abstract

The research was designed to use computed tomography (CT) with 3D-CT reconstruction imaging techniques and the various anatomical sections—*plana transversalia*, *frontalis*, and *dorsalia*—to describe the anatomical architecture of the Zebu cattle head. Our study used nine mature heads. The CT bone window created detailed images of cranial bones, mandibles, teeth, and hyoid bones. All of the head cavities were evaluated, including the cranial, orbital, oral, auricular, and nasal cavities with their paranasal and conchal sinuses. The *septum nasi*, attached to the vomer and maxillary bones, did not reach the nasal cavity floor caudally at the level of the second premolar teeth, resulting in a single median channel from the choanae to the nasopharynx. The positions, boundaries, and connections of the paranasal sinuses were clearly identified. There were four nasal conchal sinuses (that were named the dorsal, middle, ethmoidal, and ventral) and five paranasal sinuses that were described as the following: *sinus frontalis*, *maxillaris*, *palatinorum*, and *lacrimalis*, as defined in the different anatomical sections and computed tomographic images. The complicated *sinus frontalis* caused the pneumatization of all bones that surrounded the cranial cavity, with the exception of the ethmoidal and body of basisphenoid bones. The *sinus maxillaris* was connected to the *sinus lacrimalis* and *palatinorum* through the maxillolacrimal and palatomaxillary openings, and to the middle nasal meatus through the nasomaxillary opening. Our findings provide a detailed anatomical knowledge for disease diagnosis to internal medicine veterinarians and surgeons by offering a comprehensive atlas of the Zebu cattle anatomy.

## Introduction

Zebu cattle (*Bos Taurus indicus*) are a subspecies that belonged to Genus *Bos Taurus Bos* and the family *Bovidae* and is also known as the ‘humped cattle’. The Zebu were distinguished by a fatty hump in front of their shoulder region, which they developed as they adapted for tropical environments and high temperatures [1, 2]. Zebu cattle produce very little milk and are bred primarily for meat [2].

The anatomical superficial landmarks of the characteristic features of the skull have been correlated to genetic and environmental factors, which may be useful in evaluating the wide variation in phenotyping between and within breeds or species [3, 4]. A comprehensive understanding of the skull anatomy can be a useful tool in ontogenetic research and in identifying sexual polymorphisms [5, 6]. It has always been challenging to determine how various components of the head’s intricate design and its structures are interconnected [7].

Imaging techniques can help to solve this issue and advance our understanding of the anatomy of the head. Each of these approaches has advantages and disadvantages, so it will be employed for particular things. Radiography is a poor method for distinguishing between bony structures in pathological or normal situations due to the superimposition (overlapping) of bony components and cavities [8]. On the other hand, computed tomography (CT) scanning techniques offer comprehensive *plana transversalia* sectional images of the head, allowing for better differentiation between bony structures and abnormalities.

Computed tomography (CT) techniques have been extensively utilized over the past decade to diagnose a variety of disorders in both humans and animals [9]. Compared to conventional radiography, computed tomography (CT) may be a trustworthy and non-intrusive technique for assessing various medical conditions or disorders inside the head area [7]. Even though the integration of imaging techniques and stereotypical techniques has limited interest in veterinary medicine, it is currently extensively utilized to explain the normal anatomical features of the head as well as various body parts in other kinds of animals [10]. These techniques have proven to be valuable in providing detailed and accurate information about the structures and functions of the head, allowing for better diagnosis and treatment of medical conditions in animals. Additionally, the use of imaging techniques and stereological procedures in veterinary medicine has the potential to enhance our understanding of comparative anatomy and improve our ability to identify and treat diseases in different species [3]. The primary benefit of CT applications was the absence of overlapping and increased visualization of the key components of the investigated animals’ skulls [11].

There were some studies that described the anatomical features of the nasal cavity (*Cavum nasi*) with their paranasal sinuses and the cranial cavity *(Cavitas cranii)* in different animals, including large ruminants such as the buffalo [12, 13], camel [14, 15], deer [16], giraffe [17], goat [18], sheep [5], horse [19], dog [20], and cat [21]. Studies revealed variations in nasal cavity structure among species, revealing adaptations for olfaction, respiration, and vocalization. Understanding these differences can aid veterinary medicine, comparative anatomy research, and evolutionary biology studies.

The authors acknowledge that an updated, unambiguous anatomical reference for the studied species is not available, but the anatomy of closely related species has been thoroughly described, and several references are currently reported on the heads [22]. Our work was prepared to give valuable insights into the anatomical characterizations and explain the landmarks of the head of Zebu Cattle (*Bos Taurus indicus*), including their cavities and paranasal sinuses. The utilization of CT imaging, gross anatomical-section images, and three-dimensional (3D-CT) reconstruction techniques allowed for a comprehensive understanding of these structures. Such knowledge can greatly assist in clinical decision-making and enhance the accuracy of diagnostic procedures in veterinary practice.

## Materials and methods

### Collection and preparation of specimens

In the present study, twelve heads of adult healthy and clinically normal Zebu cattle (*Bos Taurus indicus, Linnaeus, 1758*) with ages ranging from 12 to 18 months and 400 to 500 kg were used. The dentation was used to determine the age of each head, according to WC Graham and MA Price [23]. The Zebu cattle were slaughtered for human consumption, not for experimental study objectives. These heads were obtained from a semiautomatic slaughterhouse of the Egyptian army, Fayoum governorate (Egypt). The Zebu Cattle were examined clinically by the veterinarian of the slaughterhouse before their slaughter to ensure that the heads were free from any musculoskeletal disorders or abnormalities. The heads were extracted from the atlas vertebrae level. The collected samples were cleansed with tap water, cooled, and prepared for gross sectional and CT anatomical techniques. The samples were then kept frozen at -20 °C and left to solidify until being processed for sectional anatomy at different planes. The *Nomina Anatomica Veterinaria* [24] served as the basis for the anatomical nomenclature of the head landmarks and components.

The present study was designed based on the guidelines for the use and care of animals of the Institutional Animal Care and Use Ethics Committee of Scientific Research (IACUC), Damanhur University, under research project number (DMU/VetMed-2023/037). The present study was approved by the local Animal Welfare and Ethics Committee, Faculty of Veterinary Medicine, Alexandria University, Egypt.

### Computed tomography scans

Within two hours of the slaughtering, the heads were utilized right away for the CT estimations. The collected heads were positioned on their ventral surfaces on the scanning table, and tomograms were made of the transverse (*plana transversalia*), frontal or dorsal (*plana dorsalia*), and sagittal sections (*plana sagittalia*) serially using the Hitachi CT scanner (CT-W455-10 A, Hitachi, Japan). Sections transverse (*plana transversalia*), lateral, and frontal sections were seen. Starting at the level of the external occipital protuberance and moving rostrally until the incisor teeth were positioned rostrally, the transverse images were scanned. The used slice thickness for each view was one centimeter, with an one-centimeter CT scan interval. General diagnostic CT system (Siemens Healthiness CT device; scanning conditions: 140 kV, 50 mA, 4 s, window width: WW: 400; window level: WL 60 Hounsfield units) set for soft windows. A bone window was obtained after changing settings (window width, WW: 1,500; window level, WL: 300 Hounsfield units). Three-dimensional CT imaging was achieved by applying the same CT system after adjusting its preferences (window width, WW: 332 window level, WL: 287 Hounsfield units). For documentation, the scanned CT images were printed via a CT digital printer, and the digital system images were stored on a hard drive for independent investigation utilizing Radiant DICOM Reader software, version 2020.2.3.

### Gross anatomical sections (or planes)

The heads used in this technique must be frozen at – 20 C to give a better cut section.

### Transverse sections or planes (***plana transversalia***)

Four frozen heads used in the CT scan technique were used for the gross transverse (cross) sectional anatomy. These frozen specimens were placed on a table with an electrical band saw, and several serial transverse sections (*plana transversalia*) with a 2 cm interval were cut starting from the level of the atlas vertebrae and continuing rostrally until about the rostral part of the oral and nasal cavity level.

### Sagittal sections or planes (***plana sagittalia***)

Four frozen heads were cut into sagittal sections (*Plana* sagittalia) at about the midline of the specimen and one paramedian section (*Plana paramediana*).

### Coronal or dorsal (***Plana dorsalia***) sections or Plana

Four frozen heads were cut on the coronal or dorsal (*Plana dorsalia*) section into three sections. Attempts were made to slice each specimen exactly according to the chosen axis.

### Preparation of the computed tomography (CT) and transverse (***Plana transversalia***) sectional images

The collected anatomical-section slices were identified, numbered, and carefully cleaned with a soft toothbrush soaked in water, left for a while to dry, and then photographed using Canon (*EOS 2000-D 18-55-IS, 24.1-MP, DSLR digital camera*), and then selected surfaces were faced towards the camera, i.e., rostral surfaces of transverse sections or planes (*plana transversalia*), right surfaces of sagittal sections or planes (*plana sagittalia*), and dorsal surfaces of the frontal or dorsal (*plana dorsalia*) sections or planes [5]. The head and paranasal components of the zebu cattle were described anatomically and through CT scanning using the following references [5, 22]. Important anatomical structures were identified in each section and corresponded to their comparable structures on corresponding soft and bone CT window images. Closely matched CT and anatomical views were selected and labeled. The slices were photographed using a digital camera (*EOS-2000D 18–55 IS, 24.1 MP, DSLR digital camera*), and the selected surfaces were facing toward the camera [3].

The obtained anatomical sections or planes, CT sections or planes, and three-dimensional CT images were analyzed, defined, and labeled utilizing Adobe Photoshop CC (Adobe System, 2022, San Jose, CA, USA).

## Results

The computed tomography (CT) imaging technique demonstrated excellent performance with its bone and soft windows. With the help of the gross sectional anatomical technique [transverse (*plana transversalia*), sagittal (*plana sagittalia*), and coronal or dorsal (*plana dorsalia*)], the computed tomography (CT) imaging technique gave clear images of the bony window of the various cranial cavities of the head as well as the gross sectional anatomy, involving the scrolled spongy nasal concha (nasal turbinate) and meatuses, conchal and paranasal sinuses, ear, eye, brain, and its appendages. The bones forming the head were identified clearly in the 3D-CT.

### Head cavities

#### Nasal cavity (*Cavum nasi*)

**Fig. 1:**
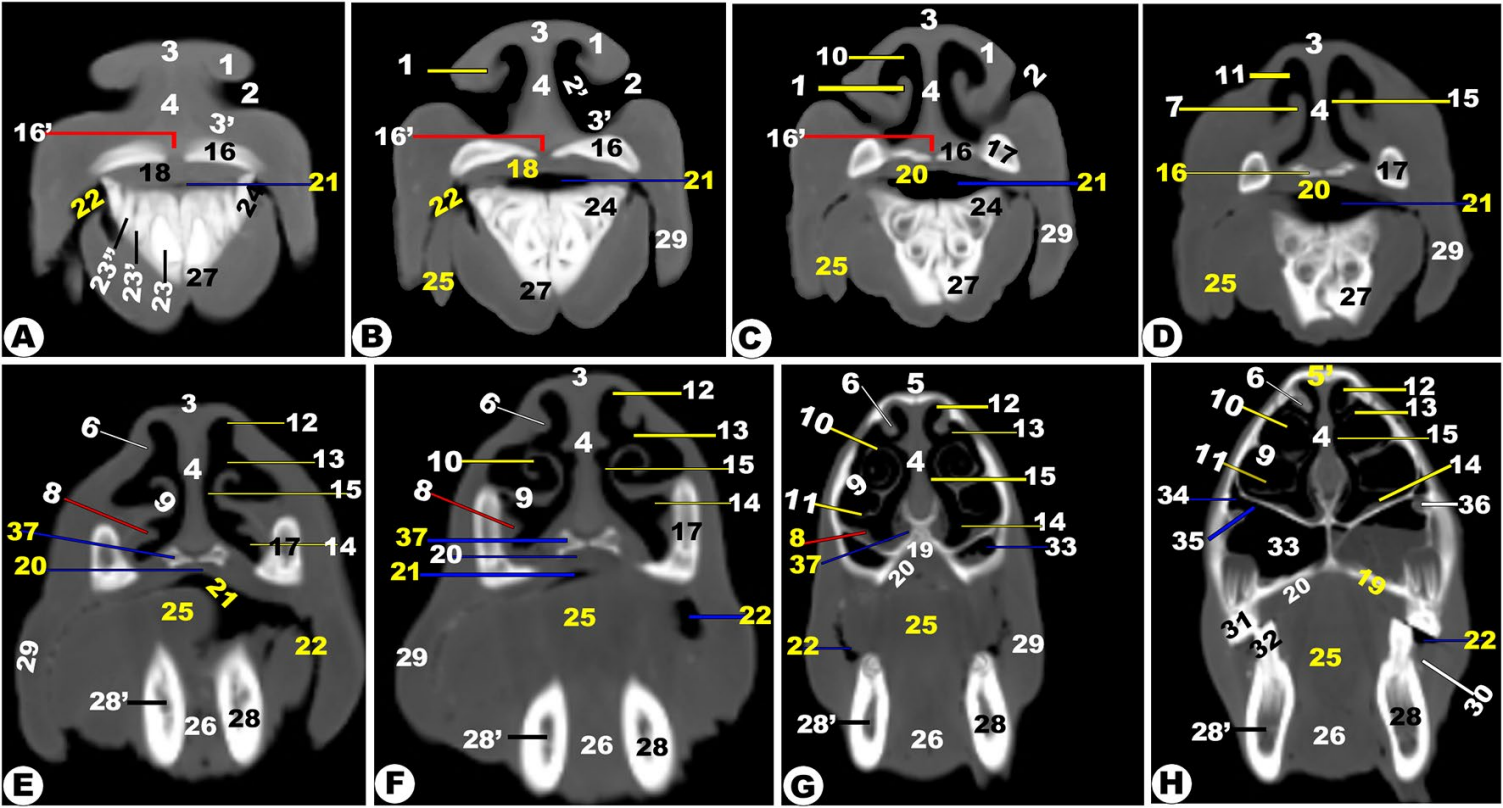
**CT images of the rostral (Views A-D) at the level of the incisive teeth and middle (Views E-H) nasal region at the level of the rostral part of the head of the Zebu Cattle from the incisor teeth to the 1**^**st**^ **upper premolar tooth.** Lateral accessory nasal cartilage (1); external nasal orifice (2); nasal vestibule (2ʹ); dorsolateral (3); and ventrolateral (3ʹ) nasal cartilage; *septum nasi* (4); incisive process (5) of nasal bone (5ʹ); dorsal nasal concha fold (6); alar fold (7), basal fold (8), dorsal spiral lamella (10), and ventral spiral lamella (11) of ventral nasal concha (9); dorsal (12), middle (13), ventral (14), and common (15) nasal meatus; Incisive bone (16); internasal fissure (16ʹ); nasal process of incisive bone (17); dental pad (18); palatine process of maxillary bone (19); hard palate (20); oral cavity proper (21); and buccal vestibule (22). 1^st^ (23), 2^nd^ (23ʹ), and 3^rd^ (23ʹʹ) lower incisive teeth; canine teeth (24); tongue (25); genioglossus muscle (26); incisive part (27) of mandible (28); mandibular canal (28ʹ); check (29); conical papillae of check (30); upper (31) and lower (32) second premolar teeth; *sinuum palatinorum* (33); *sinuum maxillaris* (34); palatomaxillary opening (35); infra-orbital canal (36); and openings of the vomeronasal organ (37)

**Fig. 2:**
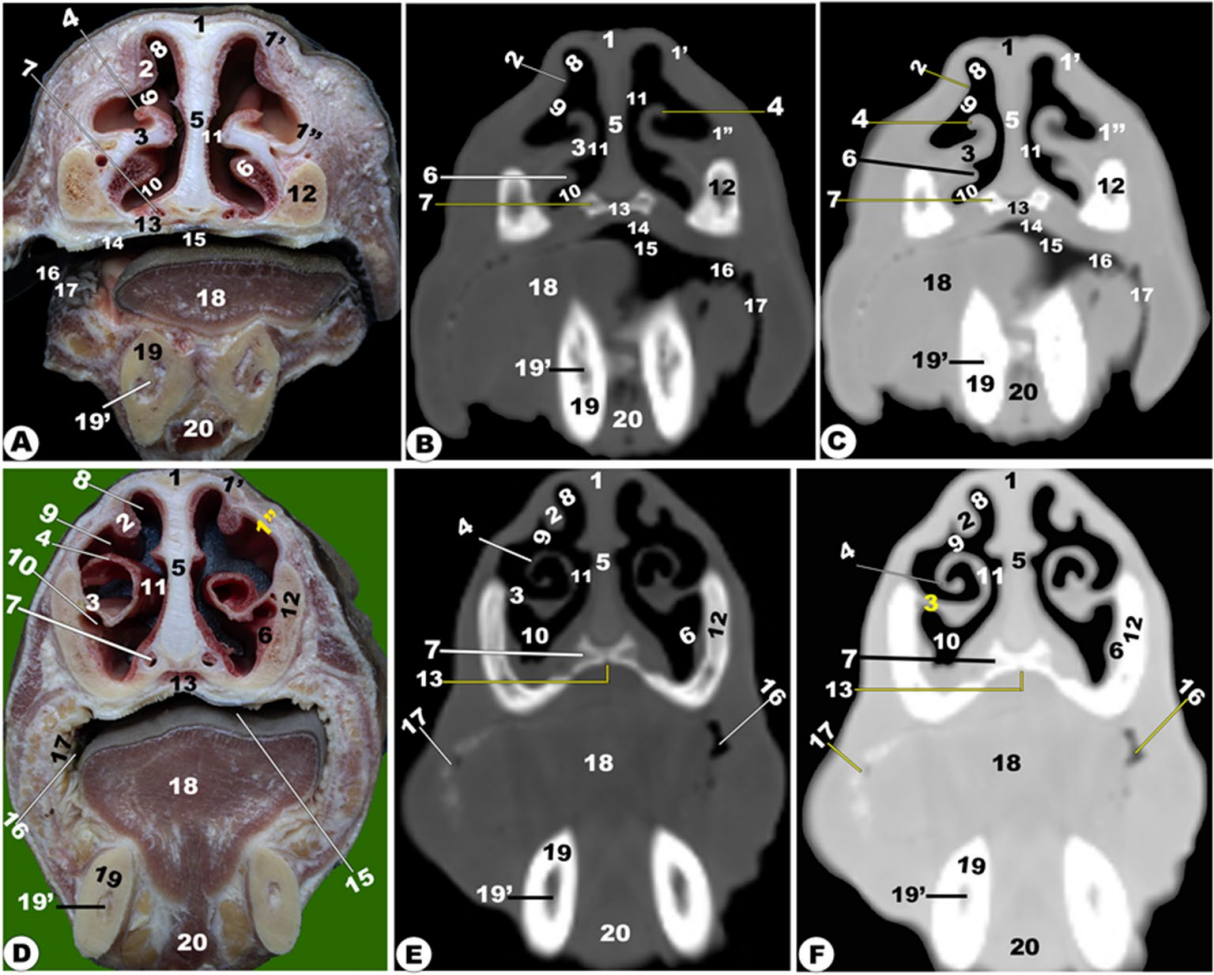
**Gross transverse (*plana transversalia*) sectional anatomical (Views A and D) and CT images of the rostral (Views B-C and E-F) at the level of the interdental space rostral to the 1**^**st**^ **upper premolar tooth.** dorsolateral nasal cartilage (1); alar cartilage lamina (1ʹ); cornu of alar cartilage (1ʹʹ); dorsal (2) and ventral (2) nasal concha; dorsal spiral lamella of ventral nasal concha (4); *septum nasi* (5); basal fold of ventral nasal concha (6); openings of the vomeronasal organ (7); dorsal (8), middle (9), ventral (10), and common (11) nasal meatus; nasal process of incisive bone (12); palatine process of maxillary bone (13); hard palate (14); oral cavity proper (15); and buccal vestibule (16) of the oral cavity; conical papillae of the check (17); tongue (18); mandible (19); mandibular canal (19); and genioglossus muscle (20)

**Fig. 3:**
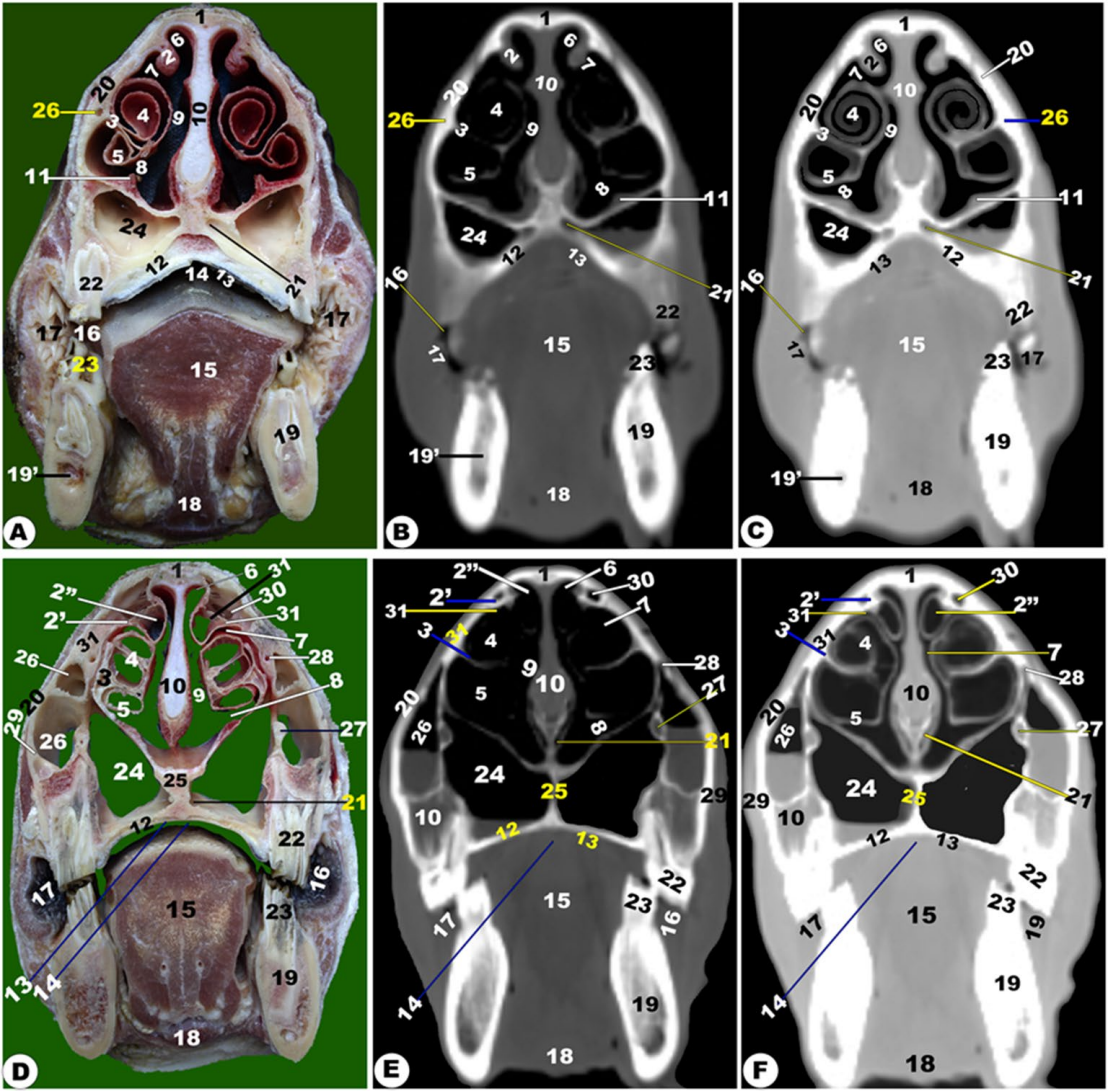
**Gross transverse (*plana transversalia*) sectional anatomical (Views A and D) and CT images of the rostral (Views B-C and E-F) at the level of the 1**^**st**^ **upper premolar tooth (A-C) and 2**^**nd**^ **upper premolar (D-F).** Nasal bone (1); *sinus frontalis* (1ʹ); dorsal nasal concha fold (2); dorsal nasal concha (2ʹ) and sinus (2ʹʹ); dorsal spiral (4) and ventral spiral (5) lamella of ventral nasal concha (3); dorsal (6), middle (7), ventral (8), and common (9); *septum nasi* (10); basal fold of ventral nasal concha (11); palatine process of maxillary bone (12); hard palate (13); oral cavity proper (14); tongue (15); buccal vestibule of the oral cavity (16); Conical Papillae of check (17); genioglossus muscle (18); mandible (19); mandibular canal (19); maxillary bone (20); openings of vomeronasal organ (21); upper first premolar teeth (22); lower first premolar teeth (23); *sinuum palatinorum* (24); interpalatine bony septum (25); *sinus maxillaris* (26); infra-orbital canal (27); nasolacrimal duct (28); facial tuberosity (29); *sinus frontalis rostralis* (30); and *conchofrontalis Ostium* (31)

**Fig. 4:**
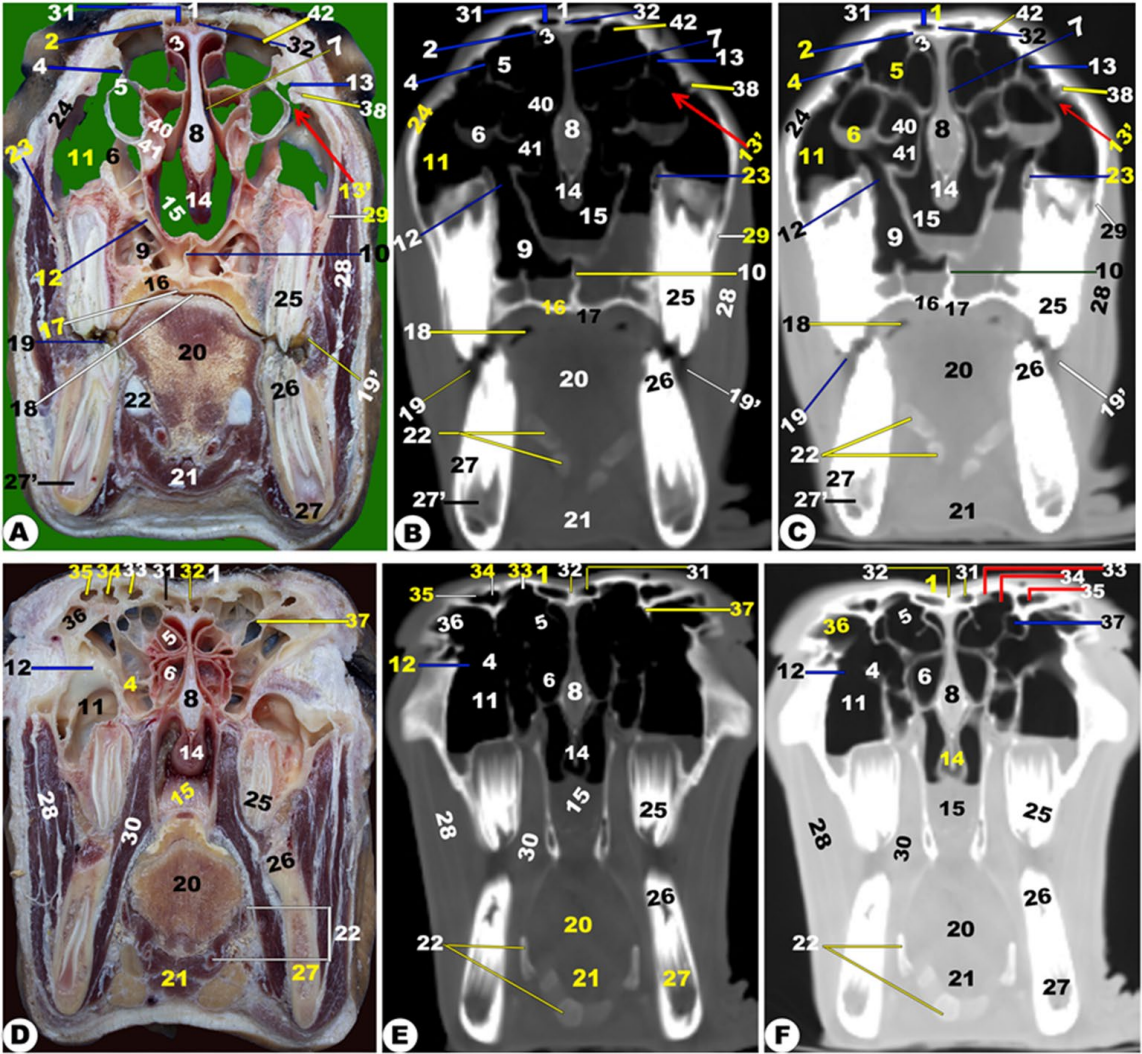
**Gross transverse (*plana transversalia*) sectional anatomical (Views A and D) and CT images of the rostral (Views B-C and E-F) at the level of the 3**^**rd**^ **upper premolar tooth (A-C) and 1**^**st**^ **molar tooth (D-F).** Frontal bone (1); dorsal nasal concha (2) and sinus (3); dorsal spiral (5) and ventral spiral (6) lamella of ventral nasal concha (4); common nasal meatus (7); *septum nasi* (8); *sinuum palatinorum* (9); inter-palatine septum (10); maxillary bone (11); maxillopalatine opening (12); *Sinus lacrimalis* (13); maxillolacrimal opening (13ʹ); nasal septum (14); choanae (15); palatine process of maxillary bone (16); Hard palate (17); oral cavity proper (18) and buccal vestibule (19) of the oral cavity; conical Papillae of check (19ʹ); Tongue (20); genioglossus muscle (21); Hyoid bone (22); Infra-orbital canal (23); maxillary bone (24); upper (25) and lower (26) second premolar teeth; mandible (27); mandibular canal (27ʹ); masseter muscle (28); zygomatic bone (29); medial pterygoid muscle (30); *sinus frontalis rostralis medialis* (31); *Septum sinuum inter-frontalium* (32); *sinus frontalis rostralis intermedius* (33); sinus frontalis rostralis medialis (34); *Sinus frontalis rostralis lateralis (35);* corneal diverticulum of *sinus frontalis* (36); supra-orbital canal (37); nasolacrimal duct (38); zygomatic bone (39); middle nasal concha (40); middle nasal sinus (41); *conchofrontalis Ostium* (42)

**Fig. 5:**
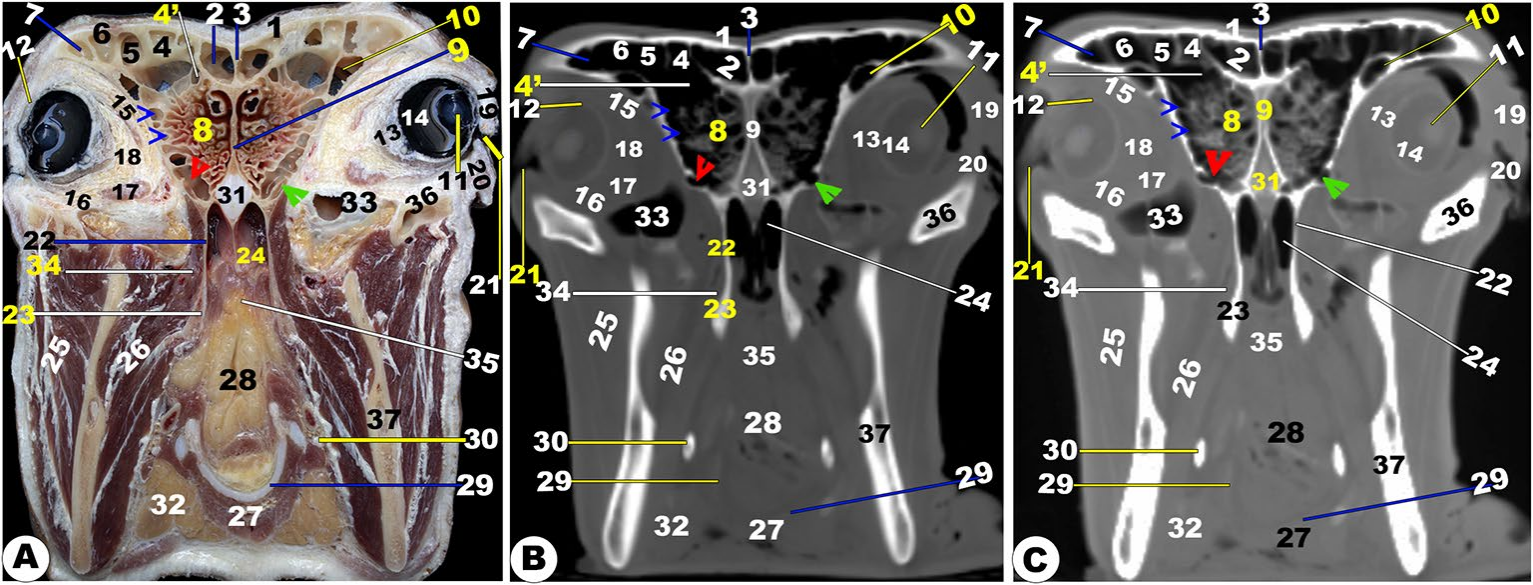
**Gross transverse (*plana transversalia*) sectional anatomical (Views A and D) and CT images of the rostral (Views B-C and E-F) at the level of the ethmoidal labyrinth caudal to the last molar tooth at the orbit of the head of the Zebu Cattle.** Frontal bone (1); *sinus frontalis rostralis medialis* (2); inter-frontal sinus (3); *sinus frontalis rostralis intermedius* (4); *sinus frontalis rostralis intermedius ostium* (4ʹ); *sinus frontalis rostralis lateralis* (5); *sinus frontalis lateralis* (6); corneal diverticulum of *sinus frontalis* (7); ethmoidal labyrinth (8); *septum nasi* (9); *sinus lacrimalis* (9); lens (11); sclera (12); choroid (13); retina (14); superior rectus muscle (15); Inferior rectus muscle (16); lateral rectus muscle (17); peri-orbital fat (18); upper eye lid (19); lower eye lid (20); orbital entrance (21); bony boundaries of choana (22); pterygoid bone (23); choana (24); masseter muscle (25); medial Pterygoid muscle (26); genioglossus and geniohyoideus muscles (27); larynx (28); thyroid cartilage (29); hyoid bone (30); presphenoid bone (31); sublingual salivary glands (32); *sinus maxillaris* (33); lateral pterygoid muscle (34); soft palate (35); temporal process of zygomatic bone (36); mandible (37). Notes: sphenoidal sinus (red arrowheads), openings of sphenoidal sinus (green arrowheads), and ethmoidal cells (blue arrowheads)

**Fig. 6:**
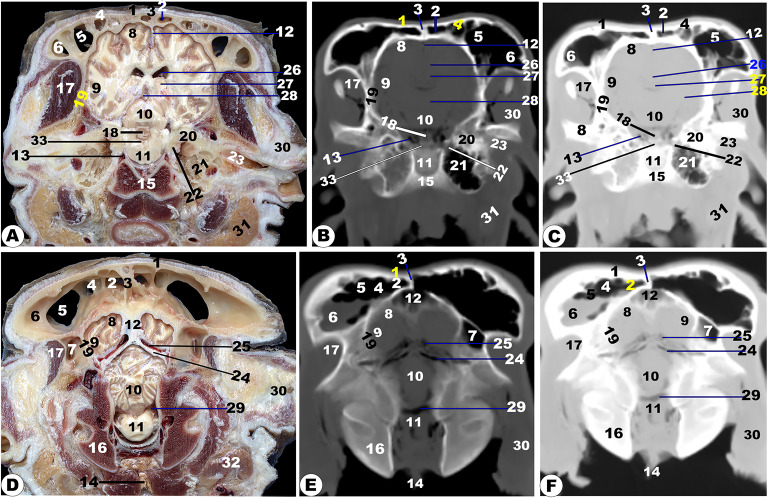
**Gross transverse (*plana transversalia*) sectional anatomical (Views A and D) and CT images of the rostral (Views B-C and E-F) at the level of the mandibular ramus of the head of the Zebu Cattle.** Frontal bone (1); *sinus frontalis rostralis medialis* (2); Inter-frontal sinus (3); *sinus frontalis rostralis intermedius* (4); *sinus frontalis rostralis lateralis* (5); *sinus frontalis lateralis* (6); corneal diverticulum of *sinus frontalis* (7); orbital diverticulum of frontal sinus (8); optic nerve (9); optic canal (9’); presphenoid bone (10); sphenoidal sinus (11); wing of basisphenoid (12); Pterygoid crest of basisphenoid (13); cerebrum (14); transverse cerebral fissure (15); dorsal sagittal sinus (16); parietal diverticulum of frontal sinus (17); choana (18); nasal septum (19); Soft palate (20); epiglottis (21); larynx (22); stylohyoid bone (23); thyroid cartilage (24); Thyroid gland (25); sternothyrohypoid muscle (26); mandible (27); masseter muscle (28); medial pterygoid muscle (29); temporal muscle (30); lateral pterygoid muscle (31); parotid lymph node (32); and lacrimal gland (33)

**Fig. 7:**
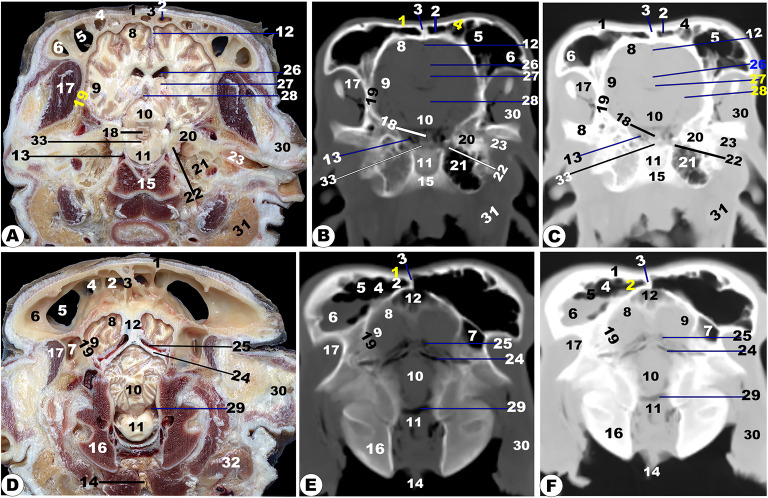
**Gross transverse (*plana transversalia*) sectional anatomical (Views A and D) and CT images of the rostral (Views B-C and E-F) at the level of the cornual process of the frontal bone, the head of the Zebu Cattle.** Frontal bone (1); *sinus frontalis caudalis median* (2); *septum sinuum inter-frontalium* (3); *sinus frontalis caudalis intermedius* (4) and *lateralis* (5); corneal diverticulum (6) and Parietal recess of frontal sinus (7); frontal (8) and parietal (9) cerebellum lobe; Cerebellar vermis (10); myelencephalon (11); flax cerebri of duramater with its dorsal (12) and ventral (13) venus sinus; spinal cord (14); Basilar part (15) and Occipital condyle (16) of occipital bone; temporal muscle (17); fourth ventricle (18); parietal bone (19); petrosal part of temporal bone (20); tympanic bulla and cavity (21); Oval foramen (22); External acoustic meatus (23); dura matter (24); transverse venous sinus (25); third ventricle (26); corpus callosum (27); hippocampus (28); pons (29); external ear (30); parotid salivary gland (31); lateral retropharyngeal lymph node (32)

**Fig. 8:**
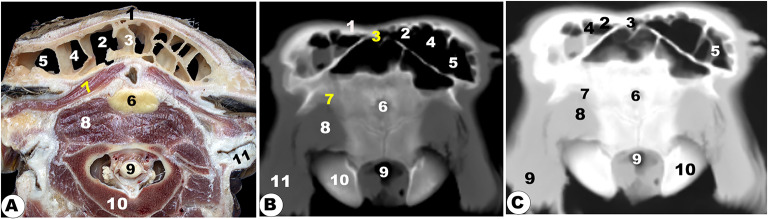
**Gross transverse (*plana transversalia*) sectional anatomical (Views A and D) and CT images of the rostral (Views B-C and E-F) at the level of the Atlas of the head of the Zebu Cattle.** Frontal bone (1); *sinus frontalis caudalis medians* (2); inter-frontal sinus (3); *sinus frontalis caudalis intermedius* (4) and *lateralis* (5); nuchal ligament (6); cervicosctularis (7); splenius muscle (8); spinal cord (9); atlas (10); external ear (11)

**Fig. 9:**
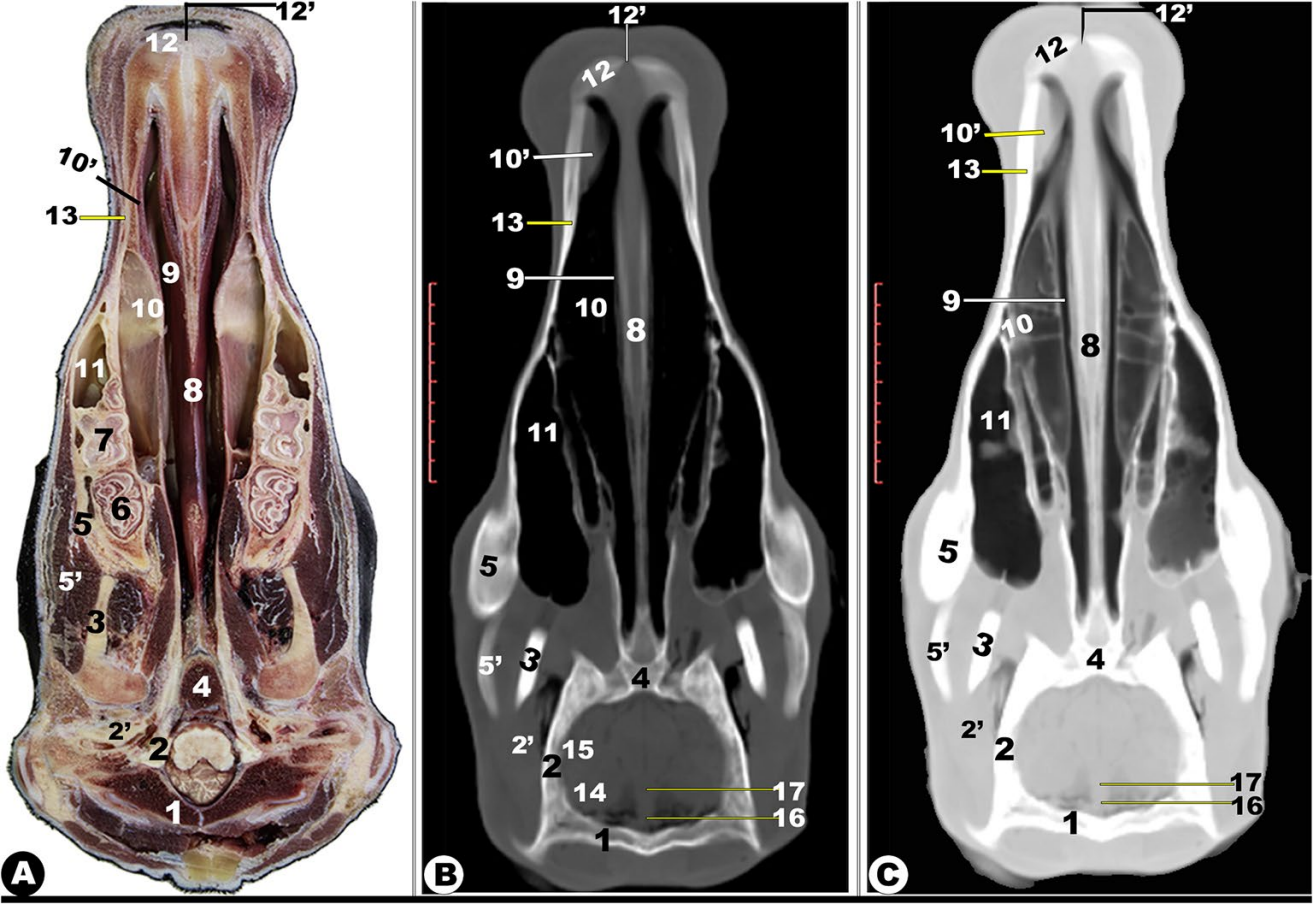
**Frontal cadaver section (View A), frontal CT section bone window (View B), and frontal CT section soft window (View C) at the level dorsal to the nasal septum of the head of the Zebu Cattle.** Occipital bone (1); temporal bone and muscle (2ʹ); lacrimal bulla (3); presphenoid bone (4); zygomatic bone (5); frontal process of zygomatic bone (5ʹ); 5^th^ (6) and 6^th^ (7) upper molar teeth; nasal septum (8); common nasal meatus (9); basal fold (10ʹ) of ventral nasal concha (10); *sinus maxillaris* (11); nasal bone (12); internasal fissure (12ʹ); maxillary bone (13); occipital (14) and temporal (15) lobe of cerebellum; flax cerebri of duramater (16); longitudinal cerebellar fissure (17)

**Fig. 10:**
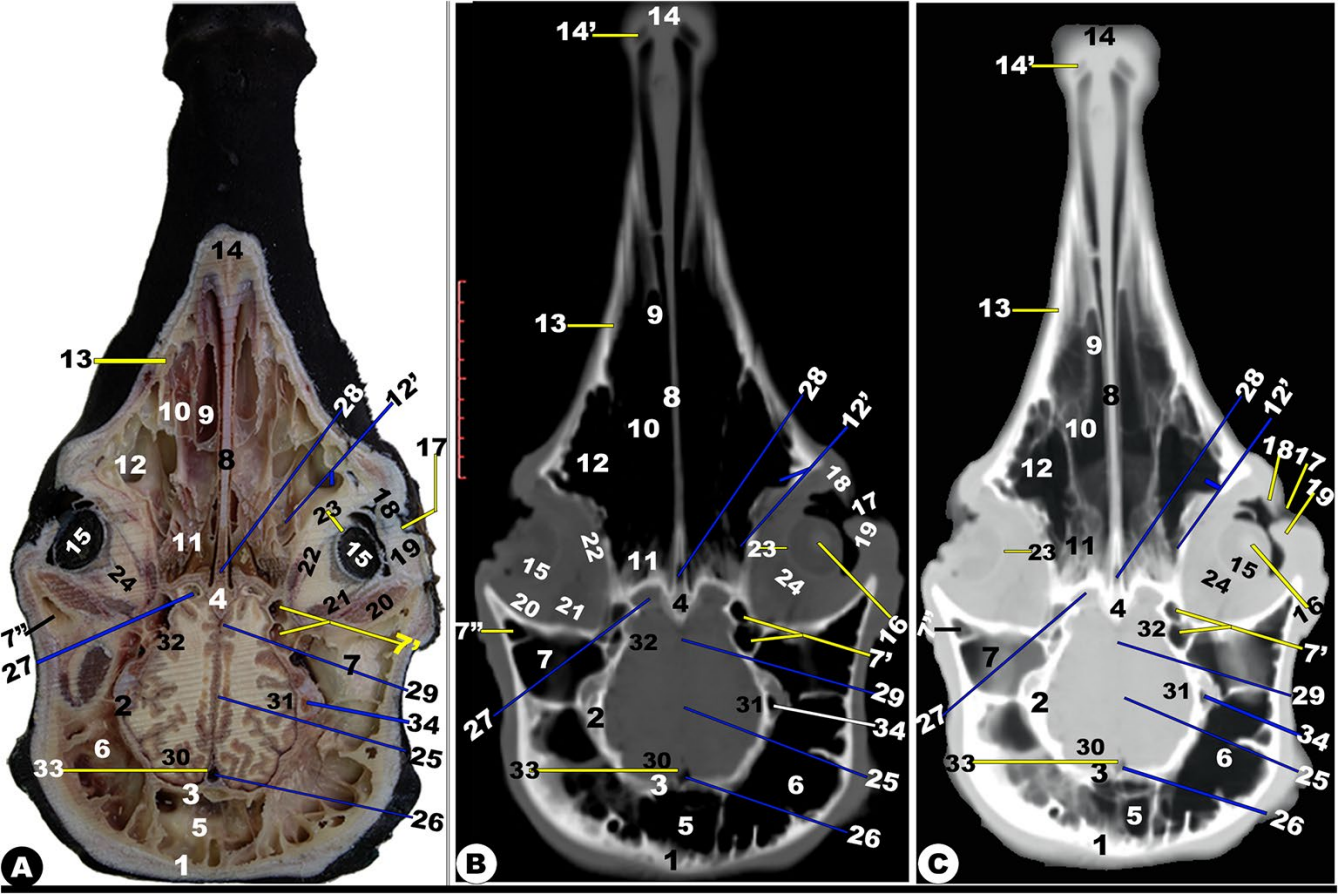
**Frontal cadaver section (View A), frontal CT section bone window (View B), and frontal CT section soft window (View C) at the level of the nasal septum and the orbit of the head of the Zebu Cattle.** Frontal bone (1); parietal bone (2) with its inner surface (3); presphenoid bone (4); *sinus frontalis caudalis medialis* (5) and *lateralis* (6); parietal (7) and temporal (7ʹ), and orbital (7ʹʹ) diverticulum of *sinus frontalis caudalis*; nasal septum (8); dorsal subdivision bulla (10) of the ventral nasal concha (9); ethmoidal nasal conchae (11); orbital diverticulum (12ʹ) of *sinus maxillaris* (12); maxillary bone (13); incisive bone (14) with its fissure (14ʹ); retina (15); lens (16); orbital fissure (17); upper (18) and lower (19) eye lid; Superior rectus muscle (20); lateral rectus muscle (21); Inferior rectus muscle (22); Sclera (23); Peri-orbital fat (24); longitudinal cerebellar fissure (25); dorsal venus sinus of duramater (26); olfactory bulb (27); sphenoidal sinus (28); ventral venus sinus of duramater (29); frontal (30), parietal (31), and temporal (32) lobe of cerebellum; flax cerebri of duramater (33); transverse venus sinus of duramater (34)

**Fig. 11:**
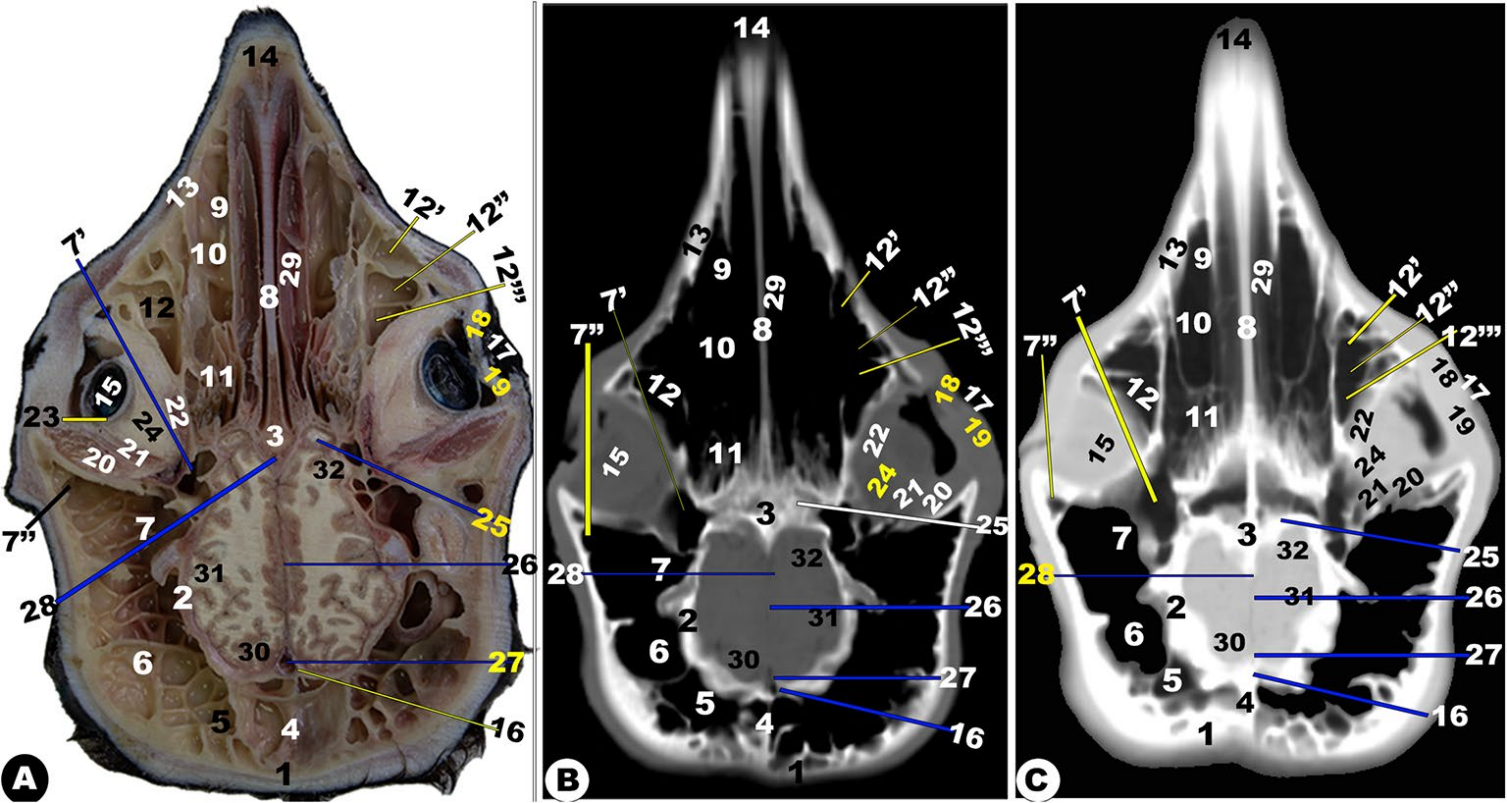
**Frontal cadaver section (View A), frontal CT section bone window (View B), and frontal CT section soft window (View C) at the level of the nasal septum and the orbit of the head of the Zebu Cattle.** Frontal (1) and parietal (2) bones; presphenoid bone (3); *septum sinuum inter-frontalium* (4); *sinus frontalis caudalis medialis* (5) and *lateralis* (6); parietal (7), temporal (7ʹ), and orbital (7ʹʹ) diverticulum of the *sinus frontalis caudalis*; nasal septum (8); subdivision bulla (10) of ventral nasal concha (9); ethmoidal nasal conchae (11); *sinus maxillaries* (12); rostral (12ʹ), middle (12ʹʹ), and caudal (12ʹʹʹ) compartments of the maxillary sinus; maxillary (13) and incisive (14) bones; retina (15); flax cerebri of duramater (16); orbital fissure (17); upper (18) and lower (19) eye lid; 20. Superior (20), lateral (21), and inferior (22) rectus muscle; Sclera (23); peri-orbital fat (24); olfactory bulb (25); longitudinal cerebellar fissure (26); dorsal (27) and ventral (28) Venus sinus of duramater; middle nasal concha (29); frontal (30), parietal (31), and temporal (32) lobe of cerebellum

**Fig. 12:**
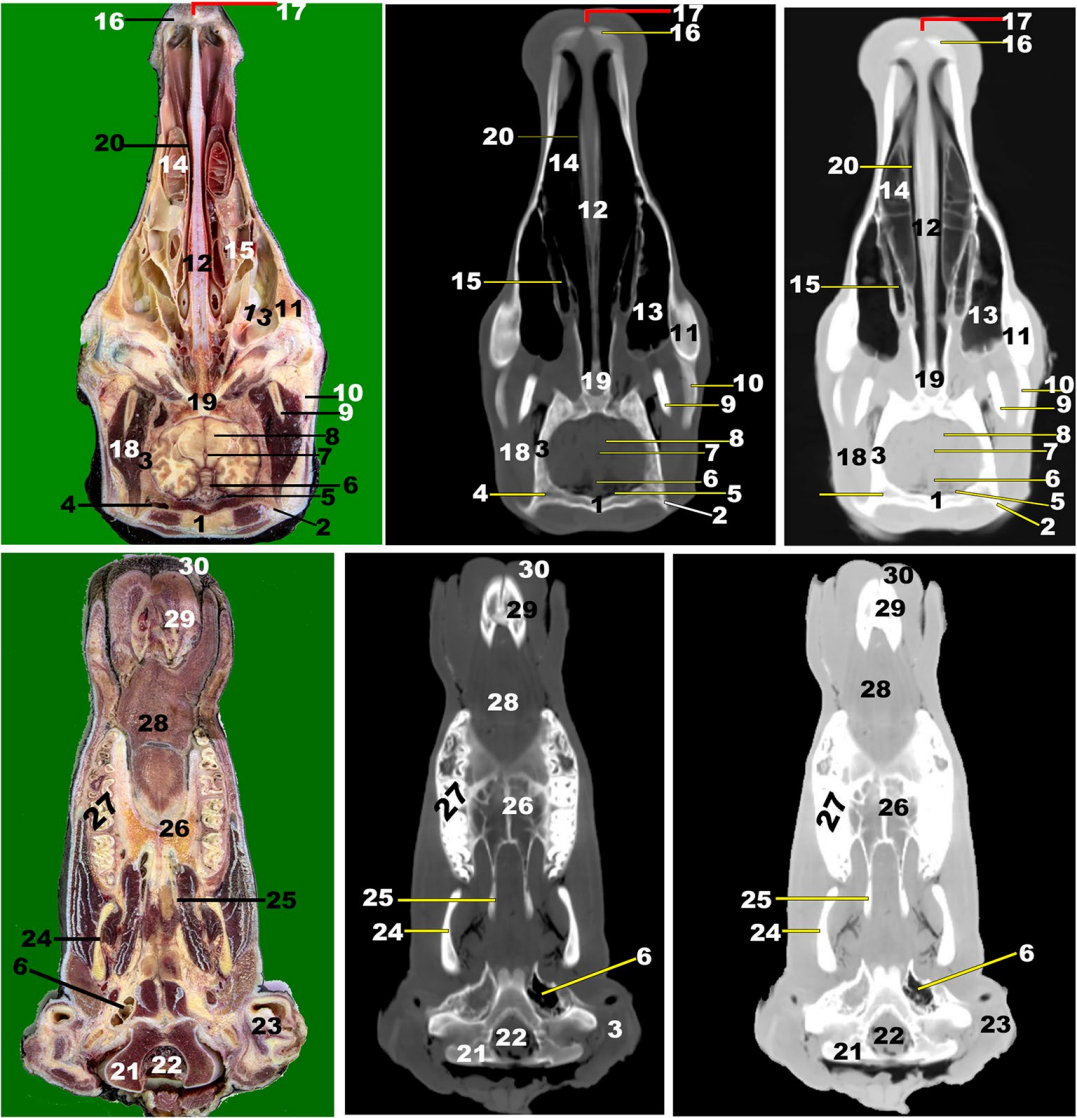
**Frontal cadaver section (View A), frontal CT section bone window (View B), and frontal CT section soft window (View C) of the head of the occipital condyle of the Zebu Cattle.** Frontal bone (1); *corneal* process (2); temporal bone (3); *sinus frontalis* (4); ventral venous sinus (5); cerebellar (6); longitudinal cerebellum fissure (7); corpus callosum (8); coronoid process of the mandible (9); orbital fat (10); maxillary tuberosity (11); nasal septum (12); *sinus maxillaris* (13); Ventral (14) and middle (15) nasal concha; incisive bone (16) with its interincissive fissure (17); temporal muscle (18); basisphenoid bone (19); common nasal septum (20); occipital condyle (21); spinal cord (22); external ear (23); coronoid process of the mandible (24); pterygoid process (25) of the *os palatinum* (26); check teeth (27); tongue (28); mandible (29); lower lip (30)

**Fig. 13:**
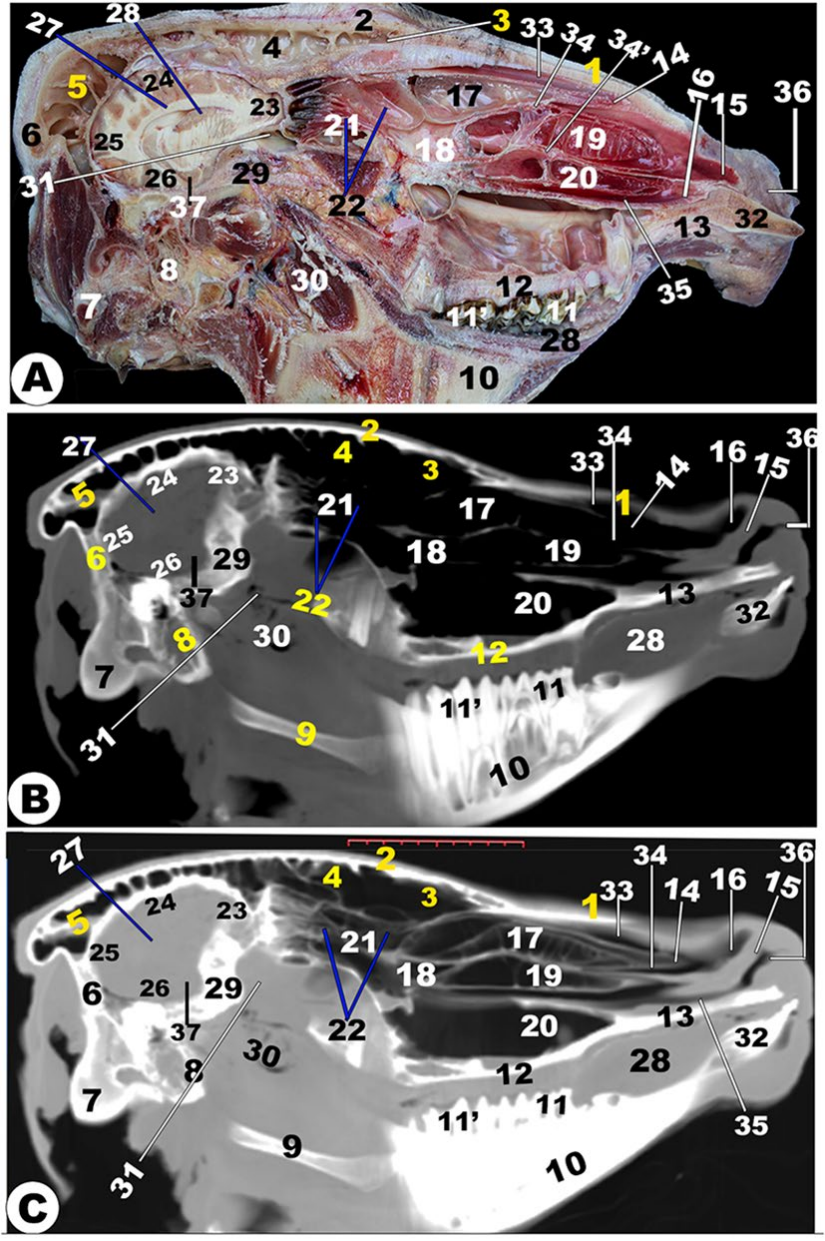
**Sagittal cadaver section (View A), Sagittal (*plana sagittalia*) CT section bone window (View B), and Sagittal CT section (*plana sagittalia*) soft window (View C) of the head of the Zebu Cattle at the level lateral to the nasal septum.** Nasal (1) and frontal (2) bones; *sinus frontalis rostralis* (3) and *caudalis* (4) with its parietal diverticulum (5); occipital bone (6) with its condyle (7); basisphenoid (8); hyoid bone (9); mandible (10); premolar (11) and molar (11ʹ) check teeth; Palatine process of maxillary bone (12); nasal process of incisive bone (13); Dorsal nasal meatus (14) with its straight fold (15); basal fold (16) of ventral nasal concha (17); middle nasal concha (18); dorsal (19) and ventral (20) spiral loop of the ventral nasal concha; ethmoidal nasal concha (21) with its nasal sinus (22); olfactory bulb (23); frontal (24), parietal (24ʹ), occipital (25), and temporal (26); corpus collassum (27); lateral ventricle (28); presphenoid bone (29); pharynx (30); Sphenoid sinus (31); incisive bone with incisive teeth (32); Dorsal nasal meatus (33); dorsal (34) and ventral (34ʹ) of the middle nasal meatus; ventral nasal meatus (35); nasal opening (36); thalamus (37)

**Fig. 14:**
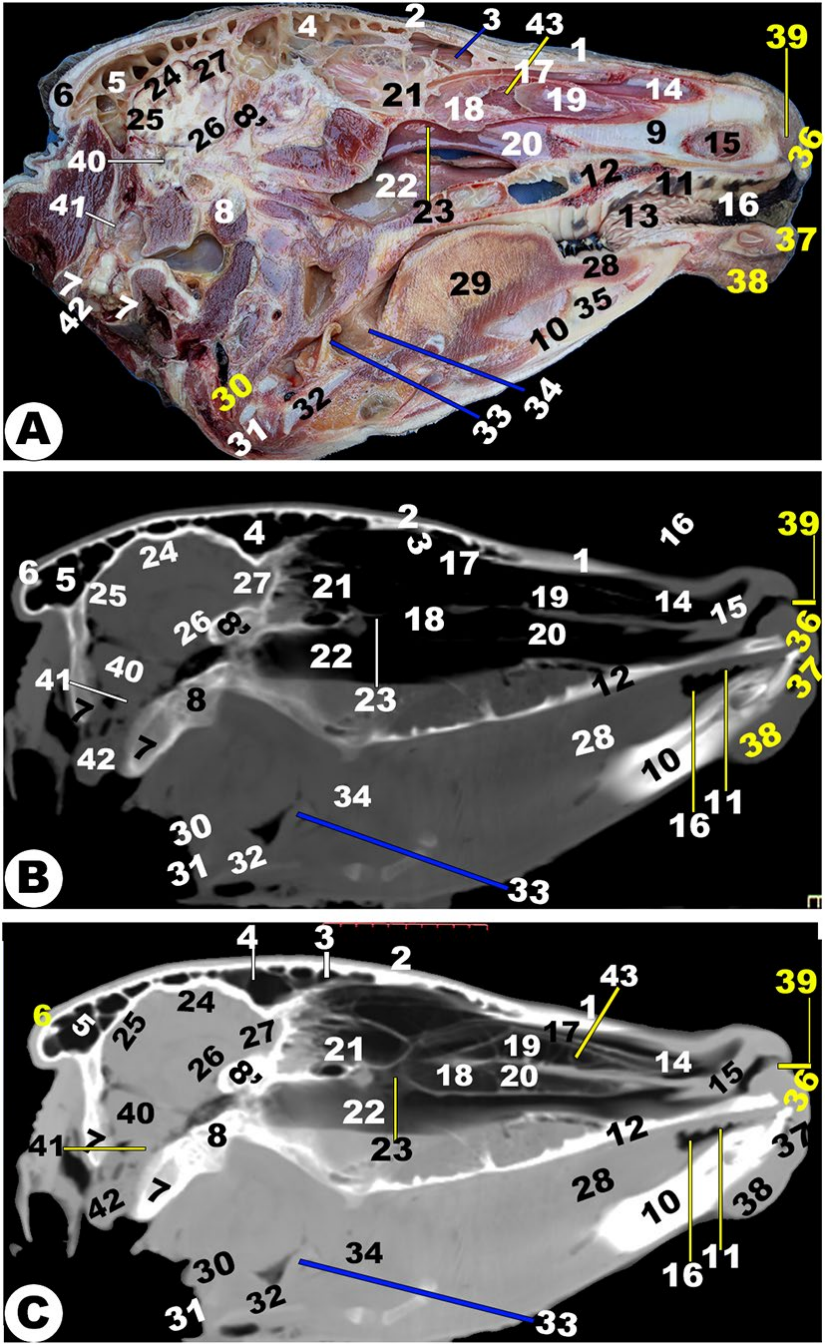
**Sagittal cadaver section (View A), sagittal (*plana sagittalia*) CT section bone window (View B), and sagittal CT section (*plana sagittalia*) soft window (View C) of the head of the Zebu Cattle at the level of the nasal septum.** Nasal bone (1); frontal bone (2) with *sinus frontalis rostralis* (3) and *caudalis* (4); Parietal diverticulum of the caudal frontal sinus (5); Occipital bone (6) with its condyle (7); basisphenoid bone (8); presphenoid (8ʹ); nasal septum (9); mandible (10); hard palate (11); palatine process of maxillary bone (12); conical papillae on inner check surface (13); straight (14) and basal (15) fold of dorsal nasal concha; Oral cavity proper (16); dorsal (17); middle (18) nasal concha; dorsal (19); and ventral (20) spiral loop of the ventral nasal concha; ethmoidal nasal concha (21); nasopharynx (22); choana (23); frontal (24), parietal (24ʹ), occipital (25), and temporal (26) lobe of cerebellum; olfactory bulb (27); tongue (28); torus lingua (29); esophagus (30); trachea (31); thyroid cartilage (32); epiglottis (33); oropharynx (34); lingual frenulum (35); upper lip (36); lower lip (37); chin (38); nasal opening (39); cerebellar (40); medulla oblongata (41); spinal cord (42); bony plates incompletely divided the dorsal nasal conchae (43)

**Fig. 15:**
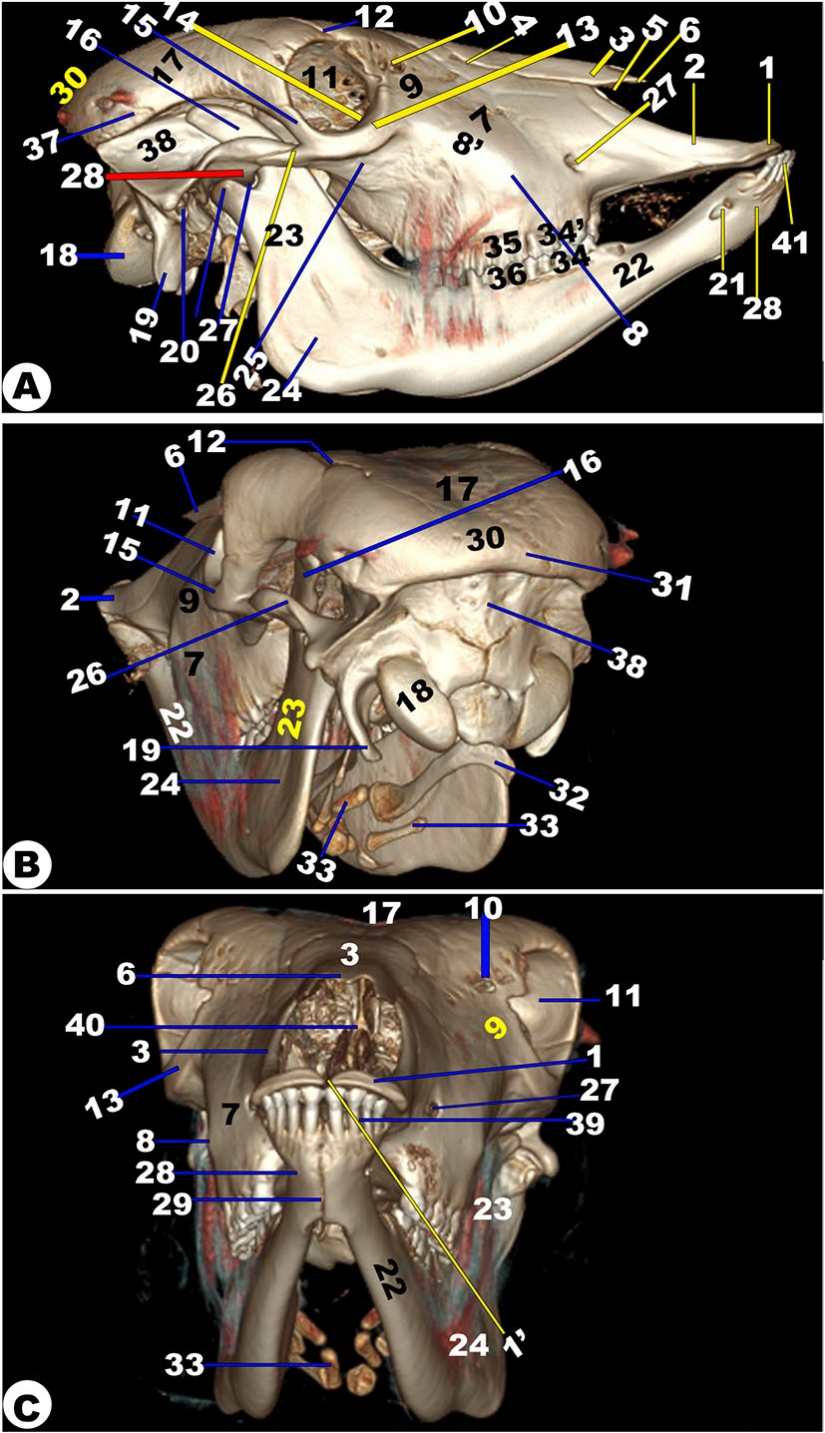
**3D of the head of the Zebu Cattle.** Incisive bone (1) with its inter-incisive fissure (1ʹ); nasal process of incisive bone (2); nasal bone (3); nasomaxillary suture (4); nasoincissive aperture (5); incisive process of nasal bone (6); maxillary bone (7) with its facial tuberosity (8) and crest (8ʹ); lacrimal bone (9); lacrimal fossa (10); orbital cavity (11); supraorbital foramen (12); orbital boundary (13); lacrimal sac fossa (14); frontal process of zygomatic bone (15); coronoid process of mandible (16); frontal bone (17); occipital condyle (18); condylar process (19); external acoustic meatus (20); mental foramen (21); body (22) and Ramus (23) of mandible; masseteric fossa (24); orbital process of maxillary bone (25); temporal process of the zygomatic bone (26); infraorbital foramen (27); mandibular suture (29); occipital bone (30); external occipital crest (31); stylohyoid (32) and thyrohyoid (33) bones; Lower (34) and upper (34ʹ) premolar; upper (36) and lower (36ʹ) molar; temporal line (37); external occipital protubernace (38); incisive teeth (39); nasal septum (40)

The nasal cavity, located between the osseous opening and cribriform ethmoidal plate, was the face portion of the respiratory conducting system. The nasal cavity (*Cavum nasi*) was identified by facial bones and consisted of the floor, lateral walls, and right and left sides, limited by the perpendicular ethmoidal plate and vomer bone. The floor and lateral walls consisted of the palatine process of the maxillary (*maxillaris*), lacrimal (*lacrimalis*), and zygomatic bones. This cavity was opened externally by the wide comma-shaped opening (*nostril*), communicated caudally with the nasopharynx through the choanae (*internal nasal opening*), and communicated with several paranasal sinuses (Figs. 1, 2, 3, 4, 5, 6, 7, 8, 9, 10, 11, 12, 13, 14 and 15). The nasal conchae (*Concha nasalis*) were located both rostral nasal cavity halves (right and left), while the ethmoidal labyrinth is located caudally (Figs. 1, 2, 3, 4, 5, 6, 7, 8, 9, 10, 11, 12, 13, 14 and 15). The common nasopharyngeal meatus, which was connected to the nasopharynx, was where the nasal cavity terminates (Figs. 4, 5 and 6). The nasal cavity (*Cavum nasi*) was identified by facial bones and consisted of the floor, lateral walls, and right and left sides, limited by the perpendicular ethmoidal plate and vomer bone. The floor and lateral walls consisted of the palatine process of the maxillary (*maxillaris*), lacrimal (*lacrimalis*), and zygomatic bones.

The different anatomical and CT section images revealed numerous cartilages that stabilized the apical nasal part, including the rostral nasal septum (*septum nasi rostralis*), dorsolateral nasal cartilage, lateral accessory cartilage, and ventrolateral nasal cartilage (Fig. 1A-F/1, 3, 3’, 4). These cartilages were found to expand from the nasal septum’s dorsal border. CT images displayed gray cartilage with intermediate attenuation in comparison to the incisive and vomer bones, while the mandible with its mandibular canal was seen with high attenuation and was more accurately identifiable with the soft tissue window in comparison to the nasal CT window setting (Fig. 1A-F).

Moreover, the nasal cavity (*Cavum nasi*) was categorized into three distinct spaces: the rostral nasal vestibular, the middle nasal cavity proper, and the caudal nasal fundus. The spaces were characterized by unique structures linked to specific functions based on their position, as depicted in (Fig. 1 to 14). The nasal vestibular part was the initial rostral nasal part, and it had three distinct nasal folds that were arranged from the dorsal to the ventral: the straight, alar, and basal folds (Fig. 1/6, 7, 8); (Fig. 2/2, 3, 6); and (Fig. 3/2, 11). The nasal cavity proper had three distinct nasal conchae, named the dorsal, the middle, and the ventral conchae, that were projected from the lateral nasal wall (Figs. 1–4/2, 2’, 3); (Figs. 9, 10 and 11/9, 10, 11); (Fig. 12/14); and (Figs. 13 and 14/17, 18, 19, 20). The nasal fundus was occupied dorsally with the ethmoidal concha and it was connected ventrally with the nasopharynx (Fig. 5/80); (Figs. 10 and 11/11); and (Figs. 13 and 14/21). The nasal bones were identified clearly in the 3D-CT, as seen in (Fig. 15/1–7).

### Nasal septum (***septum nasi***)

The median nasal septum was extended into the perpendicular ethmoidal plates to divide the nasal cavity longitudinally into two symmetric halves, right and left (Fig. 1/4); (Fig. 2/5); and (Fig. 3/10). The nasal septum was T-shaped and had a cartilaginous, flexible appearance (Figs. 4, 9, 10 and 11/8); (Figs. 5 and 13/9); and (Fig. 12/12). The nasal septum was connected dorsally with the frontal and nasal bones and ventrally with the vomer bone. The septum was attached ventrally to the groove at the median of the vomer bone in the nasal cavity and to the palatine process of the maxillary bone. However, the septum did not reach the nasal cavity floor caudally, creating a single median channel that extended from the choanae to the nasopharynx (Fig. 4/8, 14, 15) and (Fig. 5/24, 31). The vomer bone was seen on the dorsal palatine surface and maxillary bones. The nasal septum was identified clearly in the 3D-CT, as seen in (Fig. 15/40).

The vomeronasal organ (Jacobson’s organ) was located bilaterally on both sides of the cartilaginous nasal septum’s ventral part, in the nasal cavity’s rostral region. The vomeronasal organ extended from the incisive duct to the dental pad, with the 2nd or 3rd premolar tooth on each side of the nasal septum at the ventral nasal meatus (Fig. 1E-H/37); (Fig. 2/7); and (Fig. 3/21).

### Nasal conchae (***Concha nasalis***) and conchal sinuses

#### Dorsal nasal concha and sinus (***Concha and sinus nasalis dorsalis***)

It originated from the single basal lamella from the dorsal turbinate crest of the nasal bone and occupied the dorsal part of the nasal cavity (Fig. 1E-H/6) and (Figs. 2–4/2, 2’). The dorsal concha was rostrally extended in the nasal vestibule by the straight nasal fold (Fig. 1E-H/6); (Figs. 2, 3 and 4/2, 2’); (Fig. 11/10); (Fig. 12/14); and (Figs. 13 and 14/17). The dorsal concha was observed from the rostral border of the inter-alveolar space to the 2nd molar tooth level (Fig. 13/17). The caudal part of the dorsal concha, was devoid of interior sub-divisions, bullae, and cells, it was transformed into the dorsal conchal sinus, which began rostrally at the 2nd − 3rd premolar tooth level to the cribriform ethmoidal plate (Figs. 3 and 4/2, 2’); (Fig. 11/10); (Fig. 12/14); and (Figs. 13 and 14/17). The middle part of the dorsal sinus was the widest, while the two rostral and caudal ends were the narrowest. There were small bony plates that subdivided the sinus into incomplete compartments (Figs. 13 and 14/17). The dorsal conchal sinus displayed a ventrolateral extension, extending towards the nasomaxillary opening and communicating with the middle meatus, as depicted in the following figures (Figs. 3 and 4). The dorsal conchal sinus was indirectly connected to the nasal cavity by means of a wide conchofrontal (*conchofrontalis*) opening in the caudolateral wall at the 1st or 2nd molar tooth level. This indirect connection occurs through dorsal conchal sinus communication with the intermediate portion of the rostral frontal sinus (*sinus frontalis*) via a conchofrontal (*conchofrontalis*) opening (Fig. 4A-C/42); (Fig. 4D/31).

### Middle nasal concha (***concha nasalis media***) and ethmoidal sinus (sinus ethmoidalis)

It originated from the single basal lamella from the middle turbinate crest of the nasal bone and occupied the caudal part of the nasal cavity proper and the rostral part of the nasal fundus between the caudal part of the dorsal and ventral nasal conchae (Fig. 4/40); (Fig. 11/29); and (Fig. 14/18). The middle concha was extended from the 3rd premolar tooth level rostrally to the level of the caudal part of the cribriform plate caudally (Fig. 14/18). The middle nasal sinus was a small sinus that was free from any bony plates, and it was directly connected to the ethmoidal nasal meatus into the common nasal meatus. The sinus extended rostrally from the 1st molar tooth level to the rostral part of the cribriform plate caudally (Fig. 14/18).

### Ethmoidal nasal concha and sinus (***Concha and sinus nasalis ethmoidalis***)

The nasal fundus was completely filled by small, five- to seven-ethmoidal elongated projections (Fig. 5/8); (Figs. 10 and 11/11); and (Figs. 13 and 14/21). The ethmoidal concha was extended from the 2nd molar tooth level rostrally to the beginning of the brain level caudally (Figs. 13 and 14/21). The dorsal ethmoidal projection was the largest of the three. This concha contained a small sinus in each projection, with the dorsal one being the largest; additionally, from the cribriform plate, the sinus expanded rostrally and dorsally to the ethmoidal labyrinth, ending caudally at the base of the brain (Fig. 13/22). The ethmoidal sinus communicated with the fundus region on the caudal part of its roof through a large opening. The ethmoidal meatus (*Meatus nasi ethmoidalis*) was connected to the sphenoidal sinus through an opening observed medially to the caudal end.

### Ventral nasal concha and sinus (***Concha and sinus nasalis ventralis***)

It was originated from the basal lamella of the maxilloturbinate crest of the maxillary bone, which filled the ventral part of the nasal cavity (Fig. 1D-E/7, 9); (Figs. 2–3/3); and (Fig. 4/4). The ventral concha was extended rostrally in the nasal vestibule by the alar and basal folds (Fig. 1D/7); (Fig. 9/10’); and (Figs. 13 and 14/15, 16). The ventral concha was wider at its middle part at the caudal border of the 2nd molar tooth level and narrow rostrally and caudally (Fig. 9/10) and (Figs. 13 and 14/19, 20). The ventral nasal lamina was subdivided into two conchal spirals: the dorsal and the ventral spiral lamina, both of which enclosed the sinus and did not contain bullae or cells (Fig. 3/4, 5); (Fig. 4/5, 6); and (Figs. 13 and 14/19, 20). The ventral nasal concha was observed from the canine tooth level to the caudal border of the 3rd molar tooth level (Figs. 13 and 14/19, 20). The ventral conchal sinus was contained in its caudal portion by the ventral concha. This sinus was rostrally extended from the 1st premolar tooth level to the rostral border of the 3rd molar tooth level (Fig. 3/4, 5); (Fig. 4/5, 6); and (Figs. 13 and 14/19, 20). The ventral conchal sinus was connected to the middle, or ventral meatus, by a narrow opening at the 3rd premolar to the 2nd molar tooth level.

### Nasal meatuses (***Meatus Nasi***)

The nasal cavity is divided into the dorsal, ventral, and caudal meatuses by protruding nasal conchae that appear as narrow spaces (Fig. 1F-H/12, 13, 14); (Fig. 2/8, 9, 10); (Fig. 3/6, 7, 8); and (Figs. 13–14/14, 33, 35). The dorsal meatus (*Meatus nasi dorsalis*) is between the dorsal concha and the nasal bone’s internal surface; the middle meatus (*Meatus nasi medius*) is between the three nasal conchae (the dorsal, the middle, and the ventral conchae); and the ventral meatus is between the middle and the ventral nasal conchae and the floor of the nasal cavity. Moreover, the common nasal meatus (*Meatus nasi communis*) is between the protruding nasal conchae and the nasal septum (Fig. 1F-H/15); (Fig. 2/11); (Fig. 3/9); (Fig. 9/9); and (Fig. 12/20). The middle meatus was subdivided into a very narrow dorsal passage and a narrow ventral nasal passage due to the presence of the middle nasal concha (Figs. 13–14/34’, 34’’). In comparison to the dorsal and middle meatuses, the ventral nasal meatus was significantly broader and connected immediately to the choanae.

### Paranasal sinuses (***sinus paranasales***)

Our different anatomical sections and CT scan images identified the presence of the maxillary, frontal, lacrimal, palatine, and sphenoidal sinuses, as well as four conchal sinuses; the dorsal, middle, ethmoidal, and ventral nasal conchal sinuses.

### Frontal sinus (***sinus frontalis***)

It was largely excavated, completely located within the large frontal bone, and extended into the temporal, parietal, basisphenoid, interparietal, and occipital bones. The frontal sinuses were caudally extended by the small cornual diverticulum at the small cornual process and rostrally to the orbital rim, caudally to the supraorbital foramen, and rostrally to the supraorbital foramen. Within this sinus, the transverse inter-frontal septum (*septum sinuum inter-frontalium*) separated it into left and right sinuses. Each sinus is subdivided into three distinct interconnected compartments—the rostral, intermediate, and caudal frontal sinuses (*sinus frontalis caudalis*)—by bony plates.

The rostral frontal sinus (*sinus frontalis rostralis*) was divided into lateral, intermediate, and medial compartments by two sagittal oblique intra-frontal septums as described in (Fig. 3D/30); (Fig. 4/31, 32, 33, 34, 35); (Figs. 5–8/2, 3, 4, 5, 6); and (Fig. 13/3). Each compartment was subdivided into smaller compartments by oblique bony plates; additionally, each compartment contains numerous low, short bony plates or ridges, as shown in (Figs. 4–8), that were changing in dimensions and locations between the examined animals and from the right to the left sinus. The rostral frontal sinus was extended to the 3rd premolar or 1st molar teeth, with openings at the base of the rostral part and an independently opened dorsolateral into the ethmoidal nasal meatus, which appeared more obviously in CT (soft and bone windows) images in comparison to gross frontal-sections (Fig. 13/3). The caudal border of the frontal sinus was determined by the nuchal line of the occipital bone. Moreover, the medial compartment is the largest rostral frontal sinus compartment, while the lateral one is interconnected to the lateral frontal sinus (*sinus frontalis lateralis*). The lateral frontal sinus had numerous diverticulums: cornual, orbital, and parietal diverticulums (Fig. 4/36); (Figs. 5–6/7); and (Figs. 10 and 11, and 12/7, 7’, and 7’’). The dorsal conchal sinus and the dorsal portion of the lateral compartment communicate with each other.

The caudal frontal sinus was subdivided by two sagittal oblique intra-frontal septums into lateral, intermediate, and medial compartments (Figs. 7–8/2, 3, 4, 5); (Figs. 10–11/4, 5, 6); and (Figs. 13–14/5). Each compartment was subdivided into two compartments by oblique, thick bony plates (Figs. 7–8, 10–11, 13–14). The intermediate and median compartments were subdivided by a transverse oblique incomplete intra-frontal septum at the supra-orbital canal level (Fig. 4D-F). The caudal compartment was expanded toward the squamous and lateral parts of the occipital bone, connecting with the median compartment. The rostral compartment contained the postorbital diverticulum that ran between the orbital cavity and the cranial cavity (Figs. 10–12/7, 7’, and 7’’). The rostral and median compartments joined together through a large opening in the rostral portion of the supra-orbital canal, forming a common pathway as a diverticulum that ran in the rostromedial orientation. The diverticulum communicated dorsally with the ethmoidal nasal meatus by means of a narrow opening in the rostral frontal sinus. The lateral frontal sinus had numerous diverticulums: corneal, temporal, parietal, and orbital diverticulums (Fig. 4/36); (Figs. 5 and 6/7); and (Figs. 10, 11 and 12/7, 7’, and 7’’).

The supraorbital canal (*Canalis supraorbitalis*) was observed at the longitudinal interfrontal septum, between the lateral rostral frontal sinus compartment and the orbital diverticulum of the lateral frontal sinus, as described in (Fig. 4D-F/37).

### Maxillary sinus (***sinus maxillaris***)

It was situated within the triangular excavation within the maxillary and zygomatic bones and the lacrimal bulla (Fig. 3/26); (Fig. 4/11); (Fig. 5/33); (Figs. 10 and 11/12); and (Fig. 12/13). This sinus was immediately communicated with the lacrimal (*lacrimalis*) and palatine sinuses through the maxillolacrimal (*Ostium maxillolacrimalis*) and palatomaxillary (*Ostium palatomaxillaris*) openings, respectively (Fig. 4/12, 13). The nasomaxillary opening (*Ostium nasomaxillaris*), was observed between the lacrimal (*lacrimalis*) bone and the basal nasal lamina of the ventral conchae, connecting the maxillary sinus (*Sinus maxillaris*) to the middle nasal meatus. The medial of this triangular sinus was constituted by the nasal and pterygopalatine processes of the maxillary, infra-orbital canal (*Canalis infraorbitalis*), basal lamella of the ventral nasal conchae, and the nasolacrimal duct. Meanwhile, the lateral wall was made up of the inner surface of the maxillary, zygomatic, and lacrimal bones (Fig. 3/27). The rostral boundary of the sinus was observed between the projected facial tuberosity and the infra-orbital foramen (*Foramen infraorbitale*), while the caudal boundary was observed just at the lacrimal bulla in the caudodorsal margin, the maxillary tuberosity (*Tuber maxillae*) in the caudoventral margin, and the zygomatic arch in the lateral border (Figs. 10 and 11/12) and (Fig. 12/13).

The sinus was extended from the 2nd premolar tooth level to the caudal border of the 3rd molar tooth level and the facial tuberosity (Figs. 10–11/12); (Fig. 12/13). The sinus was incompletely divided into three compartments by the intra-maxillary oblique septum: the rostral, middle, and caudal compartments (Fig. 11/12’, 12’’, 12’’’). The rostral compartment represented the rostral extension of the maxillary sinus and was incompletely subdivided into two portions by incomplete bony plates. The sinus had two orbital diverticuli that were observed dorsal and ventral within the orbital cavity (Fig. 10/12’).

### Lacrimal (***lacrimalis***) sinus

It was observed inside the lacrimal bone cavity, which was located rostromedial to the orbital cavity (Fig. 4/13), and (Fig. 5/10). The lateral sinus wall was formed from the inner surface of the lacrimal bone; its medial wall was constituted by the dorsal spiral lamella of the ventral concha and ethmoidal bone; and its dorsal and caudal walls were made up of the frontal bone. The lacrimal sinus was connected with the maxillary sinus ventrally through the maxillolacrimal opening (*Ostium maxillolacrimalis*) that was observed at the 2nd or 3rd molar tooth level (Fig. 4/13’). The sinus was extended from the 2nd or 3rd molar tooth level to the cribriform plate.

It was observed inside the lacrimal bone cavity, which was located rostromedial to the orbital cavity (Fig. 4/13), and (Fig. 5/10). The lateral sinus wall was formed from the inner surface of the lacrimal bone; its medial wall was constituted by the dorsal spiral lamella of the ventral concha and ethmoidal bone; and its dorsal and caudal walls were made up of the frontal bone. The lacrimal sinus was connected with the maxillary sinus ventrally through the maxillolacrimal opening (*Ostium maxillolacrimalis*) that was observed at the 2nd or 3rd molar tooth level (Fig. 4/13’). The sinus was extended from the 2nd or 3rd molar tooth level to the cribriform plate.

### Palatine sinus (***sinuum palatinorum***)

It was situated inside the palatine bone (*os palatinum*) and the palatine process of the maxillary bone (Fig. 3/24); (Fig. 4/9). The palatine sinus was divided into two identical right and left halves by a median interpalatine septum (*septum sinuum palatinorum*), which constituted the medial wall of each division of the palatine sinus (Fig. 3/25); (Fig. 4/10). The maxillopalatine opening (*Ostium maxillopalatini*), which was shown between the second and third molar teeth, connected the palatine sinus to the maxillary sinus (*Sinus maxillaris*) directly at the infra-orbital canal level (Fig. 4/12). The sinus’ caudal part was divided into lateral and medial compartments by the palatine canal, while its dorsal wall is constituted by the maxillary and palatine bones, ventral concha, and mucous membrane, while its lateral wall was constituted by the alveolar border of the maxillary bone, the infra-orbital canal (*Canalis infraorbitalis*), as described in (Fig. 4/9). The rostral boundary was positioned rostral to the 2nd premolar tooth by 2.1 ± 0.2 cm, while the caudal boundary was positioned caudal to the 2nd molar teeth by 3.8 ± 0.9 cm.

### Sphenoidal sinus (***sinus sphenoidalis***)

It was a small, shallow cavity located within the sphenoid bone. The sinus was divided into two equal compartments (right and left) by an inter-sphenoidal septum (*Septum intersinuale sphenoidale*). The sphenoidal sinus extended to the orbital cavity’s middle part and opened directly to the ethmoidal meatus by an opening at the level of the eye’s lateral angle (Fig. 5/red arrowheads); (Fig. 6/11); (Fig. 10/28); (Fig. 13/31).

### Oral cavity (***Cavitas Oris; Cavum Oris***)

It was confined dorsally by the maxillary bone, incisive bone, hard palate, and the alveolar processes of the nasal bone, and ventrally by the incisive and molar parts of the mandible body. The oral cavity roof was formed by the hard palate rostrally and the soft palate caudally. The dental arch divided the oral cavity into two parts: the oral vestibular part (external part) and the oral cavity proper part (median internal part), with the externally located vestibular opening appearing as a U-shaped cleft (Fig. 1/21, 22); (Fig. 2/15, 16); (Fig. 3/14, 16); (Fig. 4/18, 19); and (Fig. 12/11). The oral vestibular part was divided into the labial and buccal vestibules, which latter carried numerous conical papillae on its medial wall, forming the inner surface of the check (Figs. 2 and 3/17); (Fig. 4/19); and (Figs. 13 and 14/11’).

The hard palate (*Palatum durum*) was the oral cavity roof in the rostral part, confined to the dental pad from the forward position and the inter-alveolar space laterally (Fig. 1/18); (Fig. 2/13, 14); (Fig. 3/12, 13); (Fig. 4/16, 17); and (Figs. 13 and 14/12). Following the inter-alveolar space, three premolar teeth were observed on each side, followed by three molar teeth (Fig. 1A-C/23, 23’, 23’’); (Figs. 13 and 14/32’); (Fig. 15/41). The soft palate was constituted at the terminal portion of the oral cavity, ending in the larynx, while the tongue (*Lingua*) occupied the majority of the oral cavity and extended into the oropharynx (Fig. 5/32); (Fig. 6/20); and (Fig. 12/28). The tongue was formed from three parts; the apex, body, and root; additionally, the lingual body was subdivided by the mean of the transverse lingual fossa (*fossa linguae*) into a flat rostral part and a caudal elliptical part with dorsal projected torus linguae (Fig. 1/25); (Fig. 2/18); (Fig. 3/15); (Fig. 4/20); (Figs. 5, 6, 12, 13 and 14/28); (Fig. 12D-F/8); and (Fig. 12/28). The hyoid bone (*Os hyoideum*) and mesenteric fossa were identified clearly in the 3D-CT, as seen in (Fig. 15/24, 32–33).

### Pharyngeal cavity (***Cavitas pharyngeales; Cavum pharyngeales***)

The pharyngeal cavity was divided into two distinct parts: the rostral part and the caudal part. The rostral pharyngeal part was subdivided by the projected part of the soft palate into the dorsally nasopharynx and ventrally oropharynx (*Pars oralis pharyngis*), in addition to the laryngopharynx (*Pars laryngea pharyngis*), which represented the caudal part of the pharyngeal cavity and the laryngeal entrance (Fig. 5/24); (Fig. 6A-C/18, 19); and (Fig. 14/22, 33, and 34). The CT and anatomical section images identified epiglottis, thyroid, arytenoids, and cricoid laryngeal cartilages, as seen in (Fig. 5/29); (Fig. 6/24–25); and (Fig. 13/31–35). The soft-tissue structures of the pharyngeal and laryngeal cavities, including muscles, salivary glands (*Glandulae salivariae*), and mucosal surfaces, were obvious in anatomical section images with the CT images, which were more accurately explained using the soft tissue window. The dorsally located soft palate, ventrally located tongue, and the lateral and ventrally located masticatory muscles, including temporal, masseter, and lateral pterygoideus muscles, show intermediate attenuation, while cranial bones show a high degree of attenuation (Fig. 5/25, 32, 35) and (Figs. 4 and 6/20, 24, 28–33).

### Orbital cavity (*cavitas orbitalis*)

The orbital cavity was larger than the eyeball because the ventrorostral part of the orbital cavity was occupied by lacrimal bullae. CT images revealed a moderate-density eye lens, while the sclera, cornea, and retina were clearly visible (Fig. 5/11, 12, 13, 14); (Fig. 6/9); and (Figs. 10 and 11/15, 16, 23). The orbital cavity’s walls consisted of frontal, zygomatic bones, lacrimal, the basisphenoid, and the temporal bone’s zygomatic process (Fig. 15/10, 11, 12). The rostro-medial wall was made up of the lacrimal bone (Fig. 15). The 3D-CT images provided a comprehensive views of the eyelids (*Palpebrae*), palpebral fissure, and eye canthi, as well as the vitreous body, anterior chamber, and eye muscles, as shown in (Fig. 5/19, 20, 21) and (Fig. 6/9). The peri-orbital fat was observed around the orbit with its extra-ocular muscles, while the extra-ocular muscles, including the four rectus muscles (that were named the dorsal, ventral, lateral, and medial rectus muscles) and the two oblique muscles (that were named the dorsal and ventral oblique), were clearly visible (Fig. 5/15, 16, 17); (Fig. 6/9); and (Figs. 10 and 11/20, 21, 22, 24). The lacrimal gland (*Glandula lacrimalis*) was observed to have a flattened structure on the dorsolateral orbital surface (Fig. 6/33). All the bones forming the orbital cavity (*cavitas orbitalis*) were identified clearly in the 3D-CT, as seen in (Fig. 15/9–15).

### Cranial cavity (***cavitas cranii***)

The images clarified the parts of the brain (*Encephalon*) from the olfactory bulb (*Bulbus olfactorius*) to the *myelencephalon* part. The brain’s anatomy and its important associated structures have been assessed and labeled based on their location and tissue attenuation characteristics. This study allowed for the identification of the following bones: the orbital part and squamous part of the frontal bone; the temporal bone with its squamous; the parietal; the petrosal; and the tympanic parts; the body and wing of the basisphenoid; the body and wing of the presphenoid; and the occipital bone with its squamous, basilar, and lateral parts, as seen in (Figs. 6, 7, 9, 10, 11, 12, 13 and 14). All the bones forming the cranial cavity, mandible, and mandibular joint were identified clearly in the 3D-CT, as seen in (Fig. 15).

The study utilized a bone window to create detailed images of cranial bones, mandibles, teeth, and hyoid bone, enabling clear differentiation between the cortical (excellent attenuation line) and *medullae oblongatae* (homogeneous, poor attenuation structure). However, the soft-tissue window image failed to differentiate between cortical and medulla, despite similar degrees of attenuation observed in other structures like muscles or salivary glands. The olfactory bulb, corpus callosum, pons, mesencephalon, myelencephalon, peduncle, thalamus, fornix, internal capsule, optic chiasm, cavernous sinus, cerebral hemispheres, hypothalamus, and cerebellum were among the parts of the encephalon that were revealed by CT images (Fig. 6/14–16); (Fig. 7/8–13, 18, 25, 27–29, 33); (Fig. 9/14–17); (Figs. 10 and 11/25–34); (Fig. 12A/5–8); and (Figs. 13 and 14/23–27, 37). Other noticeable structures included the third, fourth, and lateral ventricles and the mesencephalic aqueduct (Fig. 7/18, 26) and (Fig. 13/28).

The brain’s opaqueness was slightly lower than surrounding structures like head muscles and cranial bones. The olfactory bulbs were separated by a bony septum in the rostral part of the cranial cavity *(cavitas cranii)*, as depicted in (Figs. 10 and 13/27); (Fig. 11/25); and (Fig. 11/23). The cerebral hemisphere’s lobes were identified, while the corpus callosum, crescent-ventricles, and fornix were seen as less opaque regions (Fig. 6/14, 15) and (Fig. 7/7–9). The pituitary gland (*Glandula pituitaria*) and the thalamus were clear (Fig. 13/37), while the small pine-cone pineal body (*pineal body, conarium, epiphysis cerebri*) was observed in the third ventricle roof. The optic chiasm and optic nerve were also visible. The mesencephalon tectum, including the rostral and caudal colliculus, was easily visible, along with the dorsal sagittal sinus and the ventral cavernous sinus of the duramater (Fig. 6/16); (Fig. 7/12, 13, 25); (Fig. 10/26, 29, 34); (Fig. 11/27–28); and (Fig. 12/5). The occipital condyles (*Condylus occipitalis*) identified the cerebellum, pons, medulla oblongata, and cerebellum, while the bone window showed the frontal, parietal, occipital, pre-sphenoid, and basisphenoid bones that were surrounding the cranium, as seen in the following figures (Fig. 5/1, 31); (Fig. 6/1, 10–13); (Fig. 7/1); (Figs. 15–16, 9–10/1, 4); (Fig. 11/1, 3); (Fig. 12/1, 21); (Figs. 13 and 14/1, 6–8, 29); (Fig. 14/1, 8, 8’); and (Fig. 15/1, 18).

### Auricular cavity (***cavitas auriculae***)

The ear (*Auris*), a vestibule-cochlear organ (*Organum vestibulocochleare*), comprises auditory and balance organs and is described as consisting of three distinct subdivisions: the external, the middle, and the internal ear (*Auris interna, media, and externa*). The vestibular system, a balance organ, was located in the internal ear and formed from a distal cartilaginous and proximal osseous part, starting from the auricular cartilage. The eardrum was a component of the external acoustic meatus, comprising the tympanic cavity with three auditory ossicles and the auditory tube. The tympanic cavity was located within the petrosal part of the temporal bone, while the ventral hypotympanicum is an enlarged bulbous enlargement. CT images revealed the auricular cavity’s structures, including the external ear canal and cartilage, the tympanic membrane in the soft tissue window, the middle ear, petrosal temporal bone parts, and the three auditory ossicles that were named the malleus, incus, and stapes (Fig. 7/23, 30); (Fig. 8/11); and (Fig. 12/23).

### Anatomic three-dimensional reconstruction CT (3DR-CT) imaging technique of the head and skull (Fig. 15)

The nasal bone’s (*Os nasale*) and the incisor bone’s (*Os incisivum*) osseous nasal aperture (*Apertura nasi ossea*), which together formed a small nasoincisive notch (*Nasoincisiva Incisura*), served as the virtual rostral limit. The nasal bone appeared slightly straight, ending with a pointed nasal process joining the incisive bone’s nasal process, forming the naso-incissive notch at the rostral border of the premolar teeth level. The maxillary bone displayed the prominent facial crest and projected tuberosity. The supraorbital foramen was located at the medial side of the frontal bone’s border and extended rostrally towards the nasal bone, while the wide infra-orbital foramen was situated on the maxillary bone’s rostral part at the caudal border of the premolar teeth level. The mental foramen (*Foramen mentale*) was oval, and the slightly orbital fossa had a lacrimal fossa (*Fossa sacci lacrimalis*) on the lacrimal bone’s facial surface and two shallow lacrimal notches on the orbital cavity’s rostrodorsal surface.

## Discussion

The Zebu cattle, a significant global animal, are underexplored due to a lack of sectional anatomy, computed tomography (CT), and 3D-CT techniques for their anatomical structure description. Only studies on the PES region of the Zebu cattle (*Bos Taurus indicus*) have been conducted [25, 26], while there is no available data on the head region of the Zebu cattle (*Bos Taurus indicus*). Further research is needed to fully understand the anatomical features of Zebu cattle, as this information is crucial for various fields such as veterinary medicine and agriculture. With advancements in imaging technology, more detailed studies can be conducted to fill the existing gaps in knowledge about their anatomy. Our study examines the head cavities and paranasal sinuses of Zebu cattle using sectional anatomy, CT anatomical and 3D-CT atlases. The research aims to enhance the understanding of Zebu cattle’s anatomical structures, enhancing veterinary medicine and surgical procedures. It uses advanced imaging techniques to create detailed visual representations for diagnosing and treating conditions in these regions. Our study analyzes head anatomical structures and their correlation with CT images, presenting the Comprehensive Atlas of Zebu cattle (*Bos Taurus indicus*) Anatomy to internal medicine veterinarians and surgeons.

CT scans with spatial resolution improve bone and soft tissue discrimination, facilitating distinguishing characteristics in-between the nasal with their paranasal sinuses (*Sinus paranasales*) and the cranial cavities (*Cavitas cranii; Cavum cranii*) of the following animals: sheep, buffalo, and goats, as described by [3, 6, 19, 27, 28]. The current study utilizes CT, 3D-CT, anatomical transverse (*plana transversalia*), and sagittal (*plana sagittalia*) sections (planes) imaging techniques to study the anatomy of various body parts, including the head region, the nasal cavity with their paranasal sinuses, the oral cavity, the pharyngeal cavity, the auricular cavity *(cavitas auriculae)*, the orbital cavity (*cavitas orbitalis*), and the cranial cavity *(cavitas cranii)*. As reference points, we utilized the teeth, the nearby bones, and the muscles. The head, containing the brain and vital sense organs, plays a crucial role in the body. The nasal cavity also plays a thermoregulatory function, modifying the characteristics of breathed air before it enters the respiratory system. This is achieved by means of the mucosa’s extremely vascular surface, which is heated and moistened through tears and nasal secretions [29].

Our observations of the T-shaped cartilaginous nasal septum (*septum nasi*), which connects to the frontal, nasal, and vomer bones, form a single median channel from the choanae (*posterior nasal opening or internal nostrils*) to the nasopharynx, as it fails to reach the nasal cavity floor caudally. The nasal septum fails to reach the caudal part of the nasal cavity and forms a single median channel that continues to the nasopharynx. This has been observed in buffalo, camels, cattle, and donkeys [12, 15, 30], respectively. Functionally, the cartilaginous nasal septum provides structural support to the nose, helping to maintain its shape and prevent collapse during breathing.

The vomeronasal organ (*Organum vomeronasale*), also known as the Jacobson’s organ, is present in various animal species and plays a crucial role in odor detection. It is located on the right-cephalic side of the nasal septum and is harmonious in nature. In the examined animal, the nasal cavity’s rostral region contained the bilateral location of the vomeronasal organ, which was located on both sides of the ventral portion of the cartilaginous T-shaped nasal septum, extending from the incisive duct caudally to the dental pad to the 2nd or 3rd premolar tooth on both sides of the cartilaginous nasal septum at the ventral nasal meatus, similar to that described in Egyptian sheep [5] and the goat [31]. However, there are variations in the caudal extension of this vomeronasal organ in which it was observed to the lower 1st premolar tooth level [4] in Ile de France sheep; to the 2nd upper molar tooth level in the canine [32]; sheep [33]; One-humped Camel [3]; and to the 1st or 2nd premolar tooth level in buffalo [34]. The symmetrical vomeronasal organ is found on the right-cephalic side of the nasal septum and functions as an extra site for olfactory perception [35].

The position, extension, communications, and surrounding structures of the paranasal sinuses are crucial for interpreting upper respiratory tract abnormalities. Our described anatomical sections and CT scan images identify the presence of the maxillary, frontal, lacrimal, palatine, and sphenoid; additionally, the presence of four conchal sinuses, named the dorsal, middle, ethmoidal, and ventral nasal conchal sinuses. So, understanding the precise location and connections of these sinuses is essential for diagnosing and treating conditions such as sinusitis or nasal polyps. There are some differences in the extension, position, number, and openings of the paranasal sinuses between different animal species, as summarized by [3, 18, 19, 22].

Our anatomical sections or planes [transverse (*plana transversalia*), sagittal (*plana sagittalia*), coronal (*dorsal*)] and CT imaging technique reveal that there are four conchal nasal sinuses in the Zebu cattle, mainly within the caudal part of the four nasal conchae: the dorsal, middle, ethmoidal, and ventral nasal conchae, identical to what is explained in some ruminants as the cattle [22, 36], sheep [5], huemul deer [37], and goat [38]; additionally, the non-ruminant species include the horse [39, 40], and pig [36]. In contrast, some ruminant species lack the ventral conchal sinus and they are replaced by their subdivision interiorly by bullae and cells, resulting in the dorsal and ventral spiral lamella as in Egyptian water buffalos, camels, Egyptian sheep, huemul deer (*Hippocamelus bisulcus*), and le de France sheep [3, 4, 37, 41, 42].

In the examined animal, the dorsal conchal sinus begins rostrally at the 2nd to 3rd premolar teeth level to the cribriform ethmoidal plate. Meanwhile, there is some anatomical variation in the position of this sinus, such as it extending from 3rd premolar to 3rd molar tooth in buffalo [41], from 2nd to 3rd molar teeth in camels [3], from the cribriform plate to 1st or 2nd molar teeth in goats [6], from the cribriform plate to 3rd premolar to 1st molar or from 1st to 3rd molar teeth in sheep [42], and from the nasolacrimal fissure’s rostral vertex to the nasal cavity’s caudal wall in the huemul deer [37]. The current study describes that the dorsal conchal sinus communicates with the intermediate compartment of the rostral frontal sinus (*sinus frontalis rostralis intermedius*) by a wide conchofrontal (*conchofrontalis*) opening in the caudolateral wall at the 1st or 2nd molar teeth level, similar to that described in the Holstein cow [22], buffalo [41], and Egyptian goat [42], contrary to some earlier reports [3, 5, 6, 36, 43]. Moreover, [22] reported that there is a small variation in the position of the conchofrontal (*conchofrontalis*) opening, which is observed at the 2nd to 3rd molar teeth in the Holstein cow. Analogously, the dorsal conchal sinus communicates with the frontal sinus (*sinus frontalis*) by a wide opening and with the middle meatus by the maxillary sinus (*sinus maxillaris*) [44]. The sinus connection with the middle nasal meatus is reported in Egyptian sheep and camels [3, 5] and with the ethmoidal nasal meatus in goats [6]. Even though the interspecific variation in the sinus’s rostral border is generally accepted to be normal, it may be extremely correlated with the animal’s age.

Our study observed that the simple middle nasal concha is observed between the 3rd premolar tooth level rostrally and the caudal part of the cribriform plate level caudally. A similar observation is described in the Holstein cow except for the rostral boundary at the 1st, 2nd, or 3rd molar teeth [22], in buffalos and goats at the 3rd molar teeth [3, 6], in sheep at the 2nd molar teeth [42], and from the middle conchal sinus, which extends from the nasal meatus bifurcation to the caudal wall of the nasal cavity in the huemul deer [37]. In the examined animal, the middle nasal sinus directly communicates with the ethmoidal meatus into the common nasal meatus, similar to that described in the Holstein cow [22]. The current work shows that the ventral nasal sinus is observed between the 1st premolar tooth levels rostrally and the rostral border of the 3rd molar tooth level caudally. The caudal sinus boundary is similar to that observed in the Holstein cow [22]. In the examined animal, the ventral conchal sinus is connected to the middle nasal meatus or ventral meatus by a small opening at the 3rd premolar tooth to the 2nd molar tooth level, similar to that described in ruminates [22, 45]. The relationship that exists between the dorsal nasal sinus and the nasal meatus in small ruminants is not well identified [6].

Our study reveals that the large, complicated frontal sinus (*sinus frontalis*) is located completely within the frontal bone and extends into various surrounding bones, including the basisphenoid, parietal, temporal, interparietal, and occipital bones; additionally, it is extended caudally by the cornual diverticulum and rostrally to the orbital rim, supraorbital foramen, and supraorbital foramen, similar to that observed in the Holstein cow [22]. Based on the previously published data, there is a variation in the topographical extension of the frontal sinus (*sinus frontalis*), in which in the rostrodorsal of the cranial cavity of the newborn calf, the frontal sinus (*sinus frontalis*) is exclusively seen in the frontal bone [46]. With the maturation of cattle [44, 45], buffalo [41], bison [47], giraffes, and warthogs [48], the sinus grows caudo-ventrally into the temporal, interparietal, parietal, and occipital bones. When it comes to camels [3], the springbok [47], the Saanen goats [6], and the Ossimi sheep and Ile de France sheep [4, 5], the frontal sinus (*sinus frontalis*) is restricted only to the frontal bone. In the Egyptian native sheep [42], one-humped camels [14], and Egyptian sheep, the frontal sinus (*sinus frontalis*) extends into the lacrimal and parietal bones, while in one-humped camels, [49] described that the frontal sinus (*sinus frontalis*) extends to the frontal and parietal bones. In various Alcelaphus species, including Ourebia ourebi, Raphicerus campestris, and Gazelle darcas [50], the disappearance of the frontal sinus (*sinus frontalis*) is noted.

The frontal sinus (*sinus frontalis*) is divided into two halves, the left and right sinuses, by the transverse inter-frontal septum (*septum sinuum inter-frontalium*), similar to that described in cattle, buffaloes, Alcelaphus species, camels, Egyptian sheep, and Ossimi sheep [3, 5, 22, 36, 41, 42, 46, 50]. Conversely, the cattle have small openings on the caudal part of the interfrontal septum (*septum sinuum inter-frontalium*) [43, 44, 51, 52]. There are some variations about the frontal sinus’ rostral end, in which [36] reported that the sinus’s rostral border of cattle is reached to the orbital cavity; [41] reported that the sinus’ rostral end of buffalo is reached to the supraorbital groove; [3] reported that the sinus’s rostral border of the camels is reached to the 3rd premolar teeth; [53] reported that the rostral border of the Baladi goats is reached to the ethmoidal region; [6] reported that in the Saanen goats it is reached to the ethmoidal region; and in the Ossimi sheep [5], it is reached to the 3rd molar teeth.

In newborn ruminants, the location of the maxillary sinus (*Sinus maxillaris*) is in the orbit’s rostral and ventral walls and extends into the molar teeth and infra-orbital foramen with age [36, 44]. Our study describes that the maxillary sinus (*Sinus maxillaris*) is situated inside the triangular excavation of the maxillary bone, the zygomatic bone, and the lacrimal bone bulla. Meanwhile, the maxillary sinus (*Sinus maxillaris*) of the Egyptian sheep is located within a small excavation of the maxillary bone and the zygomatic bone’s rostral portion [5]. Our study describes that the sinus is extended from the 2nd premolar tooth level to the caudal margin of the 3rd molar tooth and the facial tuberosity. There are other sinus extensions that have been recorded, such as: In Egyptian water buffaloes, the maxillary sinus (*Sinus maxillaris*) extends from the 2nd premolar tooth level to the 3rd molar tooth level [41]; in one-humped camels, the maxillary sinus’ rostral border extends from the 3rd premolar to the 2nd molar teeth level [54]; in small ruminants, this sinus extends from the 3rd premolar to the 2nd molar teeth level [36]; in Saanen goat, it is extended from the 2nd premolar to the 3rd molar [6]; and from the 2nd or the 3rd premolar lacrimal bulla level in Egyptian sheep [5, 42] and in the Holstein cow [22].

In the examined animal, the rostral boundary of the sinus is between the projected facial tuberosity and the infra-orbital foramen, while the caudal boundary at the lacrimal bulla is in the caudodorsal margin, the maxillary tuberosity is in the caudoventral margin, and the zygomatic arch is in the lateral border, nearly similar to that described in most ruminants [5, 6, 36, 41, 44]. Our work describes that the sinus is immediately communicated with the lacrimal (*lacrimalis*) and palatine (*palatinorum*) sinuses through the maxillolacrimal and palatomaxillary openings, respectively, and to the middle meatus via the nasomaxillary opening, analogous to those illustrated in former anatomical literature [41, 43, 44]. The position of the nasomaxillary opening has some topographic variations between the animals, in which our study reported that this opening is observed between the lacrimal bone and the free border of the basal nasal lamina of the ventral conchae; in cattle, it is observed at the 1st or 2nd molar teeth level [44]; buffalo, it is observed at the 3rd premolar to the 1st, 2nd, or 3rd molar teeth level [41]; sheep, it is observed at the 1st molar teeth [5]; at the 1st premolar to the 1st molar teeth level in small ruminants [36] and goats [55]; and from the 21st premolar tooth level to 1.3 cm caudal to the 3rd molar tooth in native Ile-de-France sheep [4] and Egyptian sheep [42].

The present work reports that the maxillary sinus (*Sinus maxillaris*) is divided incompletely by the intra-maxillary oblique septums into three compartments: the rostral, middle, and caudal compartments. Moreover, the rostral compartment is incompletely subdivided into two portions by incomplete bony plates. Meanwhile, the sinus is divided incompletely by the infra-orbital canal into two compartments: medial and lateral [6, 42] in goats; on the other hand, the maxillary sinus (*Sinus maxillaris*) is not divided and only has the dorsal compartment in its caudolateral portion in Ile de France sheep [4].

The present work reports that the small lacrimal (*lacrimalis*) sinus is observed within the lacrimal bone cavity that is located rostromedial to the orbital cavity; additionally, its lateral wall is constituted by the inner surface of the lacrimal bone, and its medial wall is constituted by the dorsal spiral lamella of the ventral nasal concha and ethmoidal bone; and its dorsal and caudal walls are constituted by the frontal bone, similar to that observed in the Holstein cow [22]; and nearly similar observations are described in numerous ruminants [4–6, 41]. Our study observed that the sinus is extended from the 2nd or 3rd molar tooth level to the cribriform plate, similar to that observed in the Holstein cow [22]. The present work reports that the palatine sinus (*sinuum palatinorum*), situated between the palatine bone (*os palatinum*) and maxillary bone, is divided by a median interpalatine septum into two halves, serving as the medial wall, similar to that observed in the Holstein cow [22].

The present work reports that the maxillopalatine opening connected the palatine sinus (*Sinuum palatinorum*) to the maxillary sinus (*Sinus maxillaris*) at the infra-orbital canal level between the 3rd premolar and 2nd molar teeth. The maxillopalatine opening is reported in other ruminants [3–6, 22, 54], and moreover, in the Holstein cow, this opening is observed from the 3rd premolar tooth to the 3rd molar tooth level [22]. Because the palatine and maxillary (*maxillaris*) sinuses communicate with a wide maxillopalatine opening, the maxillary sinus (*Sinus maxillaris*) can be used to access the palatine sinus for surgical procedures; additionally, our research suggested that if the maxillary sinus trepanation center is chosen as the halfway point of the smallest space between the projected facial tuberosity and the orbital cavity (*cavitas orbitalis*), the palatine sinus can be simply obtained [22].

The present work reported that the small, shallow sphenoid sinus is situated within the sphenoid bone and divided into two identical compartments (right and left) by an inter-sphenoidal septum. Meanwhile, in the Holstein cow, this sinus is observed within the presphenoid bone and extends toward the basisphenoid bone in four examined cows, while it appears as a bilateral diverticulum in one examined cow [22]. However, the sphenoid sinus is completely missed in sheep, goats, and 50% of examined bovines (*bovidae*) [6, 44], while [43] noted that it may appear on one side only or completely disappear due to the fat filling it. Our study added that the sphenoidal sinus is extended to the orbital cavity’s middle part and connects to the ethmoidal nasal meatus by an opening at the eye’s lateral angle.

The ethmoidal nasal cells display various small sinuses that are opened individually into the ethmoidal nasal meatus and found between the nasal fundus part and the medial orbital walls, similar to those described in ruminants [6, 22, 41, 44]. In the current study, the ethmoidal sinus is found to be extended rostrally from the level of the cribriform plate and dorsal to the level of the ethmoidal labyrinth and to the caudal beginning of the brain; additionally, it is connected to the nasal fundus region on the caudal part of its roof through a large opening, nearly similar to that observed in the Holstein cow [22].

The current computed tomography (CT) and transverse (*plana transversalia*) sectional imaging techniques provide a comprehensive illustration of the eye structures with their surrounding peri-orbital components in Zebu cattle and auricular cavities due to the little information available about them from the different ruminants except that described in Holstein cows, camels, and sheep [5, 22, 41]. These findings assist in our comprehension of the anatomical differences in ruminant species and can help in the diagnostic process and the treatment of eye and ear-related conditions in Zebu cattle. Further research is needed to compare these findings with those of other ruminant species and to explore any potential clinical implications.

Based on CT-published data, there is a neglect of the 3D-CT reconstruction technique in veterinary research, except for some recently published data [4, 5]. This neglect of the 3D-CT reconstruction technique in veterinary research is concerning, as it may hinder advancements in the field. The recently published data, however, suggests a growing interest and potential for further exploration in this area. The 3D-CT reconstruction technique has proven to be valuable in veterinary medicine, allowing for enhanced visualization and improved diagnostic accuracy. Its potential benefits in veterinary medicine cannot be overlooked, especially considering the similarities between human and animal anatomy. Therefore, it is crucial for researchers in the veterinary field to explore and utilize this technique to further enhance their understanding and improve patient care. The current 3D-CT reconstruction technique provides a comprehensive description of the head bones of the Zebu Cattle (*Bos Taurus indicus*) and their positions and relationships with each other. This technique allows for a detailed analysis of the head bones, including their shape, size, and spatial orientation. Additionally, it provides valuable insights into any potential abnormalities or deformities present in the bone structure.

## Conclusion

The study utilized CT with 3D-CT reconstruction imaging techniques and various anatomical sections to describe the typical anatomical architecture of the Zebu cattle head. The CT bone window provided detailed images of head cavities, their bones, and their associated structures. The *septum nasi* formed a single channel in the caudal nasal part as it failed to reach the floor. The paranasal sinuses, consisting of four nasal conchal sinuses and five paranasal sinuses, they were identified through anatomical sections and computed tomographic images. The *sinus frontalis* caused the pneumatization of all bones surrounding the cranial cavity, except for the ethmoidal and body of basisphenoid bones. The *sinus maxillaris* is connected to the *sinus lacrimalis* and *palatinorum* via the maxillolacrimal and palatomaxillary openings and to the middle nasal meatus via the nasomaxillary opening.

## Data Availability

The datasets used and/or analyzed during the current study are available from the corresponding author on reasonable request.
